# Interventions Including Smart Technology Compared With Face-to-face Physical Activity Interventions in Older Adults: Systematic Review and Meta-analysis

**DOI:** 10.2196/36134

**Published:** 2022-10-31

**Authors:** Cassandra D'Amore, Julie C Reid, Matthew Chan, Samuel Fan, Amanda Huang, Jonathan Louie, Andy Tran, Stephanie Chauvin, Marla K Beauchamp

**Affiliations:** 1 School of Rehabilitation Science McMaster University Hamilton, ON Canada; 2 Department of Physical Therapy University of Toronto Toronto, ON Canada; 3 Department of Medicine McMaster University Hamilton, ON Canada; 4 Research Institute at St Joseph's Healthcare Hamilton, ON Canada

**Keywords:** aging, exercise, mobile health, mHealth, wearables, mobile phone

## Abstract

**Background:**

This is a systematic review of randomized controlled trials and a meta-analysis comparing smart technology with face-to-face physical activity (PA) interventions in community-dwelling older adults (mean age 60 years).

**Objective:**

This study aims to determine the effect of interventions including smart technology components compared with face-to-face PA interventions on PA and physical function in older adults. The secondary outcomes are depression, anxiety, and health-related quality of life.

**Methods:**

We searched MEDLINE, Embase, CINAHL, and AMED electronic databases from inception to February 2021. Two independent reviewers screened titles, abstracts, and full texts and performed data extraction and risk of bias assessments using the Cochrane risk of bias tool. The Grading of Recommendations Assessment, Development and Evaluation was used to evaluate the quality of the evidence. We provided a narrative synthesis on all included studies and, where possible, performed meta-analyses for similar outcomes.

**Results:**

This review included 19 studies with a total of 3455 participants. Random effects meta-analyses showed that interventions with smart technology components resulted in improved step count (mean difference 1440 steps, 95% CI 500-2390) and total PA (standardized mean difference 0.17, 95% CI 0.02-0.32) compared with face-to-face alone. There was no difference between groups in terms of the measures of physical function. Smart technology alone did not show significant differences between groups in any outcome. The quality of the evidence was very low based on the Grading of Recommendations Assessment, Development and Evaluation criteria.

**Conclusions:**

Interventions that include smart technology may improve daily step counts by an average of 1440 steps in community-dwelling older adults; however, the quality of the evidence was very low. Future studies are needed to improve the certainty of these results.

**Trial Registration:**

PROSPERO CRD42020135232; https://www.crd.york.ac.uk/prospero/display_record.php?RecordID=135232

## Introduction

### Background

In 2017, the global population of adults aged ≥60 years was 962 million, more than twice the number of older adults in 1980 [1]. By 2050, it is expected that the number of older adults will double, reaching nearly 2.1 billion [1]. As the population ages, delaying the onset of illness and disability and retaining physical function are top public health priorities [2]. Physical activity (PA), defined as any bodily movement produced by skeletal muscles that requires energy expenditure [3], is one way to achieve this. However, evidence suggests that 31% of the global population does not meet the recommended levels of PA [4,5], and inactivity has been identified as a leading risk factor for mortality, accounting for >5 million global deaths annually [6]. A recent umbrella review including 24 systematic reviews reported that older adults that are physically active have a lower risk of all-cause and cardiovascular mortality, breast and prostate cancer, fractures, disabilities with activities of daily living, functional limitations, risk of falling, cognitive decline, dementia, Alzheimer disease, and depression [7]. In 2018, the World Health Organization released their global action plan on PA to combat inactivity and improve health over the next decade [8].

In late 2019, SARS-CoV-2, which causes COVID-19 disease, emerged and quickly became an international health crisis, and in March 2020, the World Health Organization declared COVID-19 a global pandemic [9]. Since then, many countries have established strict public health measures to curb the spread of the disease, including social distancing and isolation. Although these measures have the benefit of minimizing viral transmission, which is critical for older adults who are at a higher risk for more severe illness [10,11], they have also exacerbated levels of physical inactivity. A systematic review including 66 studies with nearly 87,000 participants from 26 countries reported significant declines in PA during the lockdown owing to the COVID-19 pandemic [12]. Unfortunately, older adults are also at a higher risk for consequences of inactivity, such as frailty, sarcopenia, and chronic diseases, compared with their younger counterparts [13]. These data highlight an urgent need to evaluate alternative methods of improving PA levels. Fortunately, with advancements in technology, smart technology has become an increasingly relevant and studied tool for achieving health objectives [14,15]. Smart technology interventions may represent an ideal alternative to traditional face-to-face programs as they have the potential to overcome service delivery barriers such as limited access; inconvenience of travel; absenteeism from work and family [16]; and, now importantly, minimizing unnecessary exposure to COVID-19 for those who are most at risk. The role of smart technology in improving PA in older adults warrants further evaluation both now and for informing future directions of health care delivery.

Smart technology capitalizes on communication and information technologies (eg, internet and video calls) [14] and uses different mediums, such as computers and tablets, or mobile health, which includes smartphones, wearables (eg, FitBit), and mobile apps (eg, My Fitness Pal, Samsung Health, and Apple Health) [17]. Systematic reviews have demonstrated that smart technology interventions can improve PA levels, specifically steps per day and minutes per day of moderate to vigorous PA in generally healthy older adult populations (mean age ≥55 years) [18-21]. However, there are still unanswered questions and a need for more and better evidence. For example, existing reviews have examined only specific types of smart technology [20] or included only digital PA estimates without considering participant-oriented outcomes [19]. Importantly, existing systematic reviews have not compared smart technology PA interventions with more traditional modes of PA intervention delivery (ie, face-to-face) [15,20-23]. This comparison is essential for determining whether interventions that include smart technology components are more, less, or as effective as face-to-face alone interventions. Therefore, the purpose of this review is to determine the effects of PA interventions that use smart technology compared with face-to-face PA interventions on PA and physical function in community-dwelling older adults.

### Review Question

This systematic review will answer whether the PA interventions that use smart technology are more, less, or as effective as face-to-face alone interventions for increasing PA and function in older adults. The secondary questions were as follows: (1) What are the effects of smart technology PA interventions on secondary outcomes, including health-related quality of life (HRQoL), anxiety, and depression? and (2) Does the effectiveness of smart technology interventions differ by type of PA or by the type of smart technology used (eg, wearable vs mobile app)?

## Methods

### Overview

This systematic review was conducted in accordance with a peer-reviewed protocol [24] registered in PROSPERO (CRD42020135232) and followed the PRISMA (Preferred Reporting Items for Systematic Reviews and Meta-Analysis) guidelines [25]. The full protocol has been published elsewhere [24].

### Data Source and Searches

A comprehensive search of the MEDLINE, Embase, CINAHL, and AMED databases from inception to February 2021 was conducted after consultation with a health research librarian [26]. The full search strategies are available in Multimedia Appendix 1 [27-45]; common Medical Subject Headings across databases included age, technology, physical fitness, with keywords to capture all types of PA and smart technology. Reference lists of included studies were hand searched to identify additional relevant studies.

### Study Selection

#### Overview

Two independent reviewers completed screening for both the titles and abstracts and full-text articles using the web-based referencing software system Covidence (Veritas Health Innovation). Disagreements were resolved by consensus or arbitration by a third reviewer as necessary.

#### Inclusion Criteria

We included studies that met the following criteria: (1) community-dwelling older adults with a mean age of ≥60 years [46], (2) interventions that promoted PA using smart technology, (3) face-to-face interventions in comparator groups, (4) a primary outcome measure of PA or physical function, and (5) randomized controlled trials (RCTs) published in English in a peer-reviewed journal.

#### Exclusion Criteria

We excluded studies that evaluated participants admitted to an inpatient unit in a hospital or long-term care home, interventions that only used audio phone calls (ie, with no video or SMS text messaging equivalent to the use of a landline), video games, or virtual reality. Studies that used a quasi-experimental design were also excluded.

### Data Extraction and Risk of Bias

Data from the included studies were extracted independently and in duplicate using a standardized data collection form [24]. Two reviewers independently assessed studies using the Cochrane risk of bias tool [47] and the Grading of Recommendations Assessment, Development, and Evaluation (GRADE) system [48].

### Data Synthesis and Analysis

Meta-analyses for primary and secondary outcomes were conducted using random effects models with standardized mean difference (SMD) and mean difference (MD) where appropriate in Review Manager (RevMan; version 5.4, The Cochrane Collaboration, 2020) [24]. According to the Cochrane handbook, when necessary, we converted scales to correct for the difference in direction [49], and median and IQR were converted following the methods of Wan et al [50]. When possible, we performed sensitivity analyses by removing studies with an overall rating of a high risk of bias for each outcome. This deviates from the initial protocol, wherein we planned to remove only studies with a high risk of bias in ≥3 domains. Where appropriate, we completed subgroup analyses for our secondary questions. Where possible, we completed analyses for interventions that used smart technology alone compared with face-to-face alone.

## Results

### Overview

**Figure 1:**
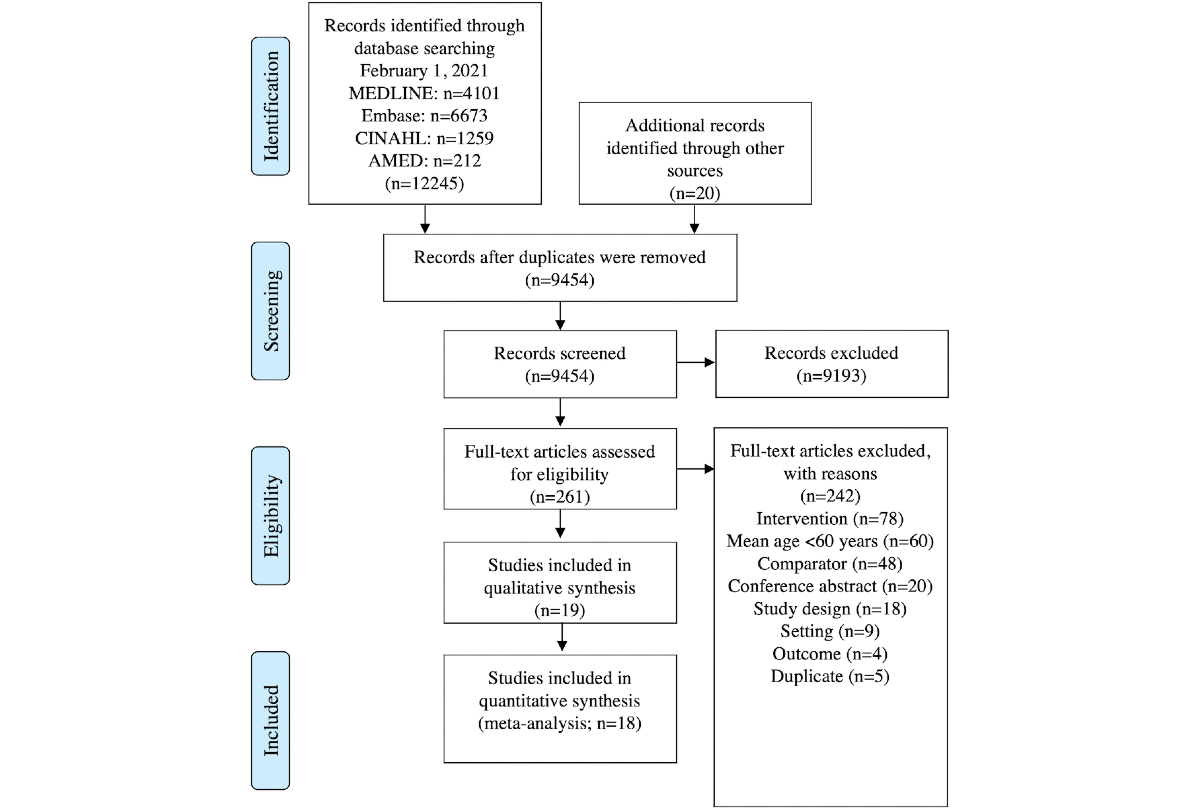
PRISMA (Preferred Reporting Items for Systematic Reviews and Meta-Analysis) flow diagram of evidence search and selection.

We identified 12,245 records from our search; reviewers screened 9434 titles and abstracts after duplicate removal, and 19 RCTs were eligible for inclusion (Figure 1). The reasons for full-text exclusion are provided in Multimedia Appendix 1. Reviewers attempted to contact 7 corresponding authors for missing information, and 1 author responded. All the studies were combined in a narrative synthesis, evaluating a total of 3455 participants. A total of 18 RCTs with 3405 participants randomized to either a smart technology or face-to-face intervention were included in the quantitative analysis.

Characteristics of the studies are shown in Tables 1-2. Of the 3455 participants, 1874 (54.24%) were female, and the mean age ranged from 60 to 72 years. Studies were conducted at outpatient or community practices in the following countries: United States (6/19, 32%); Belgium (2/19, 11%); Netherlands (2/19, 11%); United Kingdom (2/19, 11%); and one each in Australia, Chile, Denmark, Finland, Hong Kong, New Zealand, and Spain. Participants were community-dwelling older adults, with 14 studies focused on specific clinical populations, including people with chronic obstructive pulmonary disease (COPD) [27-31], cardiovascular disease [32-35], diabetes [36,37], knee arthritis [38,39], obesity [40], and cognitive impairment plus physical frailty [41].

**Table 1:** Characteristics of included studies with author names beginning with A to J.

Study and country	Population, sample size (n)	Intervention^a^	Usual care	Outcomes	Key findings
Alonso-Domínguez et al [36], 2019, Spain	T2DM^b^, median age IG^c^=60.8 (IQR 7.8) years; CG^d^=60.4 (8.4) years, 45.6% female, n=204	[Smartphone app] Daily use of mobile app for 3 months + heart-healthy 4-km walks once per week for 5 weeks	10-minute standardized counseling session on PA^e^ and healthy diet; information leaflet provided	PA: steps per day measured by pedometer; weekly PA measured by IPAQ^f^-Spanish version	Significant increase in daily steps per day (*P*<.05 at 3 and 12 months), aerobic steps (*P*<.05 at 3 months), distance walked (*P*<.05 at 3 months), and total PA (*P*<.05 at 3 months) for intervention group
Armit et al [43], 2005, Australia	Community-dwelling older adults (55-70 years), mean age 64 (SD 4.6) years, 64.9% female, n=37 recruited, 28 with 1 assessment	[Pedometer] Pedometer use for 12 weeks; counseling session; information booklet; diary for goal setting and self-monitoring; 3 follow-up phone calls to reevaluate, reinforce, and discuss adherence issues	Counseling session; information booklet; diary for goal setting and self-monitoring; 3 follow-up phone calls to reevaluate, reinforce, and discuss adherence issues	PA: self-report survey^g^	No significant difference in PA between groups at either time point
Audsley et al [44], 2020, United Kingdom	Community-dwelling older adults from Falls Management program, mean age IG=76.9 (SD 7.0) years; CG=73.8 (SD 6.4) years, 73.3% female, n=50	[Pedometer] 6 sessions of 60 to 90 minutes of motivational interviewing and behavior change techniques over 6 months; pedometer use worksheets with PA diaries + usual care	24-week group exercise program (strength, balance, cardiovascular, and flexibility exercises) + 30-minute 2 times per week home exercise program; how to get up from a fall and strategies to reduce inactivity	PA: weekly MVPA^h^ measured by Phone-FITT^i^ questionnaire	No significant differences in MVPA between groups
Barnason et al [32], 2009, United States	Postoperative CABS^j^ (aged >65 years), mean age 71.2 (SD 4.9) years, 17% female, n=280	[Telerehabilitation] Telehealth symptom management for 6 weeks on strategies to address common symptoms after CABS and improve outcomes (eg, PA and functioning) + usual care	Usual care not defined	PA: modified 7-day Activity Interview; activity counts by accelerometer; Physical Activity and Exercise Diary; quality of life: MOS SF-36^k^	Higher overall energy expenditure in usual care group, except at 3 weeks where intervention group had higher expenditure; no other significant differences
Christiansen et al [38], 2020, United States	Unilateral TKR^l^ (aged >45 years), mean age 67 (SD 7.0) years, 53.4% female, n=43	[Wearable] 6-month standard outpatient PT^m^ with a Fitbit Zip, weekly steps per day goal from a PT, 6 monthly phone calls from a research assistant	Standard outpatient PT, including printed home exercise program; 6 monthly phone calls after discharge discussing overall health	PA: steps per day and MVPA per week measured by Actigraph	Increased steps and minutes per week in MVPA for intervention group at both follow-ups
de Blok et al [27], 2006, Netherlands	COPD^n^ (aged 40-85 years), mean age IG=65 (SD 10.4) years, CG=62.5 (SD 12.3) years, 75% female, n=21	[Pedometer] 4 sessions of 30-minute exercise counseling (steps per day goal setting), pedometer feedback for 10 weeks + usual care	Conventional pulmonary rehabilitation of exercise training, dietary intervention, and psychoeducational modules over 9 weeks	PA: pedometer steps per day^g^; function: chair stand, arm curl, 8-foot up-and-go, 2-minute step test; other: Beck Depression Inventory	Significantly improved 8-foot up-and-go test and 2-minute step test for intervention group; no significant differences between groups in steps per day
Frederix et al [33], 2015, Belgium	Cardiac conditions (CAD^o^ and CHF^p^), mean age 61 (SD 8.5) years, 17% female, n=140	[Multicomponent] 24-week PA telerehabilitation; exercise training protocols + center-based cardiac rehabilitation for 12 weeks (usual care)	Center-based cardiac rehabilitation for 12 weeks (45 sessions—2 exercise sessions per week, dietary consultation, and psychologist session)	PA: CPET^q^ VO_2_^r^ peak^g^, accelerometer PA, self-report IPAQ converted to MET^s^-minutes per week of MVPA; quality of life: HeartQoL	Significant improvement in mean VO_2_ peak (*P*<.001), steps per day, MET-minutes per week of MVPA (*P*=.01), and health-related QoL^t^ (*P*=.01) for intervention group
Hansen et al [28], 2000, Denmark	COPD (no participation in pulmonary rehabilitation in the preceding 6 months), mean age 68.3 (SD 9.0) years, 55% female, n=134	[Telerehabilitation] 10-week telerehabilitation program including group-based supervised, standardized 35-minute exercise program via videoconference, followed by 20 minutes of patient education 3 times per week	10-week conventional pulmonary rehabilitation program including group-based supervised, standardized 60-minute exercise program 2 times per week in conjunction with 1 time per week education session lasting 60-90 minutes	PA: accelerometer PA; function: 6MWT^g,u^, 30-second sit-to-stand; quality of life: HADS^v^, EQ-5D^w;^ COPD: COPD assessment test and Clinical COPD Questionnaire	No significant differences in 6MWT; however, intervention group maintained improvement at 22 weeks; significantly improved HADS and depression scores (*P*<.05) and COPD Assessment Test scores (*P*=.04) in intervention group

^a^The authors grouped interventions by the type of smart technology; groupings are indicated in square brackets at the beginning of each intervention description.

^b^T2DM: type 2 diabetes mellitus.

^c^IG: intervention group.

^d^CG: control group.

^e^PA: physical activity.

^f^IPAQ: International Physical Activity Questionnaire.

^g^Primary outcomes of the individual studies.

^h^MVPA: moderate to vigorous physical activity.

^i^FITT: Frequency, intensity, type, and time

^j^CABS: coronary artery bypass surgery.

^k^MOS SF-36: Medical Outcomes Study 36-item Short Form survey.

^l^TKR: total knee replacement.

^m^PT: physiotherapy.

^n^COPD: chronic obstructive pulmonary disease.

^o^CAD: coronary artery disease.

^p^CHF: congestive heart failure

^q^CPET: cardiopulmonary exercise testing.

^r^VO_2_: maximal oxygen uptake.

^s^MET: metabolic equivalent.

^t^QoL: quality of life.

^u^6MWT: 6-minute walk test.

^v^HADS: Hospital Anxiety and Depression Scale.

^w^EQ-5D: EuroQoL 5 Dimensions.

**Table 2:** Characteristics of included studies with author names beginning with K to Z.

Study and country	Population, sample size (n)	Intervention^a^	Usual care	Outcomes	Key findings
Kawagoshi et al [29], 2015, Finland	COPD, mean age IG=74 (SD 8) years; CG=75 (SD 9) years, 11% female, n=39	[Pedometer] Pedometer + home-based pulmonary rehabilitation including breathing retraining, exercise training, respiratory training, and monthly 45-minute education sessions	Home-based pulmonary rehabilitation including breathing retraining, exercise training, respiratory muscle training, and monthly 45-minute education sessions	PA: total PA as measured by accelerometer; function: quadriceps muscle force; 6MWT; Pulmonary: Chronic Respiratory Disease Questionnaire	Significant increase in walking time (*P*=.04), dyspnea, and quadriceps force in intervention group; significant improvements in pulmonary function tests, 6-minute walk distance, and Chronic Respiratory Disease Questionnaire in both groups
King et al [42], 2020, United States	Community-dwelling adults aged 50 years, mean age 62.3 (SD 8.4) years, 78.8% female, n=245	[Telerehabilitation] 1:1 initial counseling session, pedometer use; additional (up to 28) 10 to 15 minutes counseling sessions by a virtual advisor over 12 months	1:1 initial counseling session, pedometer use; additional (up to 28) 10 to 15 minutes counseling sessions conducted by a human advisor over 12 months	PA: walking minutes per week^b^ total PA, MVPA, daily PA measured by accelerometer, self-report weekly sedentary behavior; quality of life: Vitality Plus scale	Significantly increased steps per day (*P*=.02) in intervention group; significant decreases in both groups for reported sedentary time
Kwan et al [41], 2020, Hong Kong	Mild cognitive impairment and physical frailty, mean age 71.0 (SD 9.0) years, 85% female, n=33	[Smartphone app] Smartphone apps for individualized goals, to log PA data, performance reviews, and e-reminders; communication app for e-coaching, personalization of goal settings, and messages of praise + control intervention; all interventions were for 12 weeks	Conventional behavior change techniques via PA counseling, telephone follow-up, health education, and exercise training. All interventions were for 12 weeks	PA: steps per day measured by accelerometer; MVPA and non-MVPA per week measured by accelerometer; other: Fried frailty index; MoCA^c^	Significant increase in MVPA (*P*=.04), walking time (*P*=.03), steps per day (*P*=.02), brisk walking (*P*=.009), and peak cadence (*P*=.003) for intervention group; adherence to face-to-face sessions was 100% for both groups; smartphone compliance was 54.1 (SD 1.2) days per participant (range 0-56 days)
Maddison et al [34], 2015, New Zealand	Ischemic heart disease, mean age 60.2 (SD 9.3) years, 19% female, n=171	[Multicomponent] Automated text messages for 24 weeks encouraging 30 minutes per day MVPA 5 days per week + regular exercise prescription, behavior change strategies, website access with model vignettes, self-monitoring, information	Encouraged to participate in cardiac rehabilitation typically including education sessions, psychological support, and PA encouragement and offer to join a supervised exercise club	PA: self-reported PA measured by IPAQ; other: PVO_2_^d^ assessed during CPET^b^; self-efficacy and motivation to exercise; SF-36 and EQ-5D	No significant difference in PVO_2_ between groups; significant improvements in self-report PA (*P*=.05), walking (*P*=.02), self-efficacy (*P*=.04), and health-related QoL (*P*=.03) in intervention group
Mendoza et al [30], 2020, Chile	Stable COPD, mean age 68.7 (SD 8.5) years, 39.2% female, n=102	[Pedometer] Pedometer use and steps per day goals for 3 months, 3 monthly follow-up sessions with a physician and physiotherapist to increase step count	3 monthly counseling sessions with physician and PT to increase PA, advised to walk minimum 30 minutes per day	PA: steps per day^b^ measured by pedometer; other: health status and exercise capacity	Significant increase in PA (*P*<.001) and exercise capacity (*P*=.03) in intervention group
Mouton and Cloes [45], 2015, Belgium	Community-dwelling adults (aged 50 years), mean age IG1=61.2 (SD 6.3) years; IG2=69.8 (SD 7.4) years; IG3=63.2 (SD 5.7) years; CG=66.1 (SD 6.8) years, 60% female, n=149	[Website] Three groups, all 3-month duration: (1) web-based intervention (PA promotion + monthly PA feedback); (2) center-based intervention—12 weekly sessions of group exercise; and (3) mixed intervention (web- and center-based intervention)	No intervention received	PA: self-report using IPAQ-S^e^; other: stages of change; awareness of PA; and participant acceptance of intervention	Mixed intervention increased PA level (*P*=.04); center-based intervention (*P*<.001) and mixed intervention (*P*=.01) increased PA stages of change; web-based intervention (*P*=.02) and mixed intervention (*P*<.001) increased PA awareness
Roberts et al [35], 2019, United States	Community-dwelling adults (aged 60 years) with moderate to high risk of CVD^f^ events, mean age 72 (SD 7.4) years, 60% female, n=40	[Wearable] Activity tracker + strategies to increase PA for 20 weeks; usual care (8-week center-based exercise intervention); goal to achieve 150 minutes of MVPA per week for 12 weeks; encouragement of nonexercise PA	Usual care including 2 for per week center-based exercise intervention for 8 weeks; instruction to achieve 150 minutes of MVPA per week for remaining 12 weeks; behavioral counseling to encourage nonexercise PA	PA: daily activity measured by accelerometer; function: 6MWT; 4-meter gait speed; grip strength; SPPB^g^	Significant increase in steps per day for intervention group
Tabak et al [31], 2014, Netherlands	Stable COPD, mean age IG=65.2 (SD 9.0) years; CG=67.9 (SD 5.7) years, 37% female, n=34	[Multicomponent] 4-week daily use of mobile activity coach for feedback, motivation, and target PA levels + usual care (medication and PT—weekly group training sessions)	Could consist of medication and weekly group training PT sessions	PA: steps per day^b^ measured by pedometer; COPD: Clinical COPD Questionnaire (health status); other: compliance	No significant differences in steps per day; nonsignificant improvement in health status in intervention group; 86% adhered to the activity coach
Talbot et al [39], 2003, United States	Symptomatic knee OA^h^, aged ≥60 years, mean age IG=69.6 (SD 6.7) years; CG=70.8 (SD 4.7) years, 76.5% female, n=34	[Pedometer] Pedometer use + daily step goals; education booklet on exercise and managing pain; usual care (12 sessions of 1-hour arthritis self-management education)	12 sessions of 1-hour arthritis self-management education (including a session on exercise)	PA: steps per day^b^ by pedometer, PA over time (accelerometer); function: leg muscle strength; 100-foot timed walk-turn-walk; timed stair climb; timed chair rise	23% steps per day increase for intervention group vs 15% decrease in control group; improved usual pace gait speed (*P*=.04) and isometric leg strength (21%—compared with 3.5% loss in control group)
Weinstock et al [37], 2011, United States	Diabetes mellitus, mean age 70.9 (SD 6.8) years, 63% female, n=1650	[Multicomponent] Educational videoconferencing for 4-6 weeks to review blood glucose and blood pressure measurements; pedometer use with goals set for 2 years	Usual care from PCP^i^; PA encouraged by pedometer use with goals set between participant and PCP for 2 years	PA: diabetes self-care activities for assessment of PA; other: feasibility; acceptability; and CARE Depression Instrument	Significantly slower rate of decline in PA (*P*=.01) and lower rate of PI^j^ (0.04); significantly higher PA levels (*P*<.001) in intervention group
Yates et al [40], 2009, United Kingdom	Overweight or obese (BMI ≥25), mean age 65 (SD 8) years, 34% female, n=87	[Pedometer] Pedometer use + 180-minute education session on causes and complications of impaired glucose tolerance + exercise information	Two groups: (1) Same education session as intervention but no pedometer and (2) usual care—information pamphlet	PA: steps per day measured by pedometer, self-reported walking by IPAQ, and total MVPA	Compared with usual care group 2, significant increases in steps per day, self-reported walking, and total MVPA at 3, 6, and 12 months in intervention group (all *P*<.05)

^a^The authors grouped interventions by the type of smart technology; groupings are indicated in square brackets at the beginning of each intervention description.

^b^Primary outcomes of the individual studies.

^c^MoCA: Montreal Cognitive Assessment.

^d^PVO_2_: peak oxygen uptake.

^e^IPAQ-S: International Physical Activity Questionnaire-Short.

^f^CVD: cardiovascular disease.

^g^SPPB: Short Performance Physical Battery.

^h^OA: osteoarthritis.

^i^PCP: primary care provider.

^j^PI: physical impairment.

### Interventions

Detailed descriptions of the smart technology interventions in each study are provided in Multimedia Appendix 1. A total of 16 studies included a single smart technology component, including smartphone apps [36,41], wearable activity trackers (eg, Fitbit) [35,38], telerehabilitation (eg, video conferencing, virtual advisor, or health buddy device) [28,32,42], and pedometers (ie, only provides step counts) [27,29,30,39,40,43,44], whereas the remaining 3 had multiple components, including video conference and pedometer [37]; website and SMS text messaging [34]; and website plus pedometer, SMS text messaging, and email [33]. A total of 15 studies included smart technology and a face-to-face component in the intervention group [27,29-36,38-41,43,44]. Furthermore, 4 studies evaluated smart technology alone versus face-to-face alone [28,37,42,45]. The length of interventions ranged from 10 weeks [27] to 1 year [29], and follow-ups ranged from 6 weeks [32] to 2 years [37].

### Risk of Bias

Overall, the risk of bias was a concern for studies included in this review. On the basis of the outcome with the highest risk of bias (ie, if we assessed risk of bias for 3 outcomes in a study and 1 of those was rated a high risk of bias and the other 2 were some concerns, we rated the study as high risk of bias), 14 studies were judged to be high risk [27,29,31,32,34,36,37,39-45], 4 studies had some concerns [28,30,35,38], and 1 study had a low risk of bias [33]. The areas of greatest concerns were missing outcome data (10/19, 53% high risk) and risk of bias related to measurement of the outcome (eg, awareness of intervention and influence of knowledge of intervention on the assessment; 9/19, 47% high risk). A summary figure is available in Multimedia Appendix 1.

### Physical Activity

#### Overview

**Figure 2:**
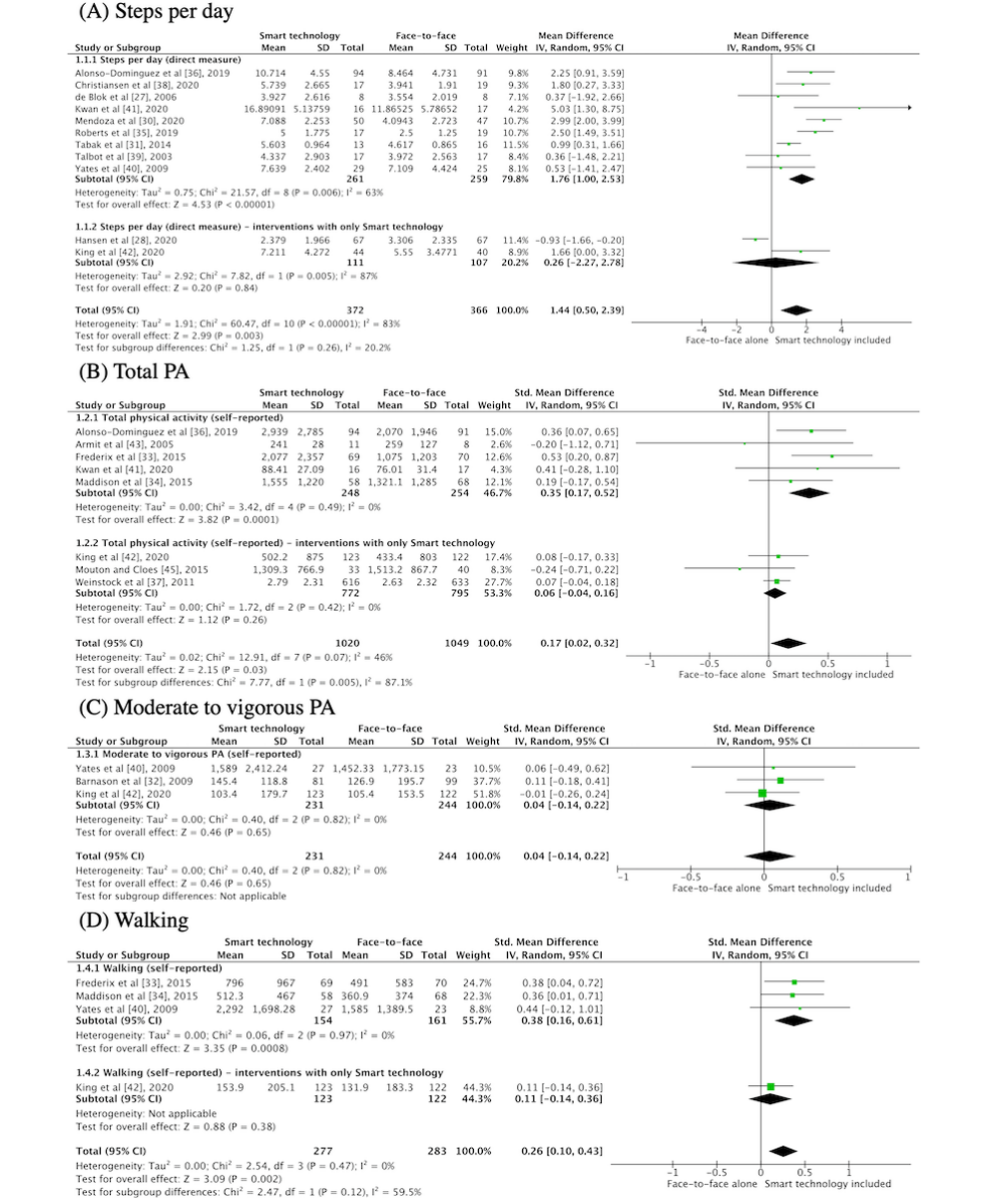
Meta-analysis of effect of smart technology versus face-to-face alone on physical activity (PA).

All included studies assessed the effect of smart technology interventions on PA [27-45]. The types of PA evaluated included steps per day [27,28,30,33,35,36,38-40,42], total PA [28,33,34,36,37,41-43,45], moderate to vigorous PA [32,38,40-42,44], and walking [29,33,34,40-42], assessed either directly (eg, with pedometer or activity tracker) or indirectly (eg, self-report measures such as the International Physical Activity Questionnaire [33,34,36,40,45], Diabetes Self-Care Activities for assessment of PA [37], Community Health Activities Model Program for Seniors PA [42], Physical Activity Scale for the Elderly [41], activity diary [32], and Active Australia Survey [43]). Studies were grouped by type of PA, and 4 meta-analyses were performed for daily step counts, total PA, moderate to vigorous PA, and walking (Figure 2).

Compared with face-to-face interventions, interventions that included smart technology improved step count, with the meta-analysis of 11 studies and 738 participants demonstrating a MD of 1440 steps (95% CI 500-2390; Figure 2) [27,28,30,31,35,36,38-42]. Smart technology also improved total PA scores (8 studies, n=2069; SMD 0.17, 95% CI 0.17-0.52) and walking (4 studies, n=560; SMD 0.26, 95% CI 0.10-0.43) compared with face-to-face interventions. The meta-analysis of 3 studies (n=475) for moderate to vigorous PA was not statistically significant (SMD 0.04, 95% CI −0.14 to 0.22). We performed sensitivity analyses for all outcomes except moderate to vigorous PA. On the basis of the results of the risk of bias for the sensitivity analyses, we removed 5 studies with high risk of bias in the steps per day; however, the remaining 6 studies still favored smart technology with a MD of 2.03 (95% CI 0.35-3.71). Only 2 studies remained with 1388 participants for total PA; the difference was no longer significant with a SMD of 0.27 (95% CI −0.18 to 0.72). Finally, our sensitivity analysis for walking containing 2 studies (n=384) was not significant with a SMD of 0.22 (95% CI −0.04 to 0.48).

#### Subgroup Analyses

##### Smart Technology Components

We completed meta-analyses for subgroups according to the type of smart technology that was used for steps per day and total PA scores (Multimedia Appendix 1, section 5). Studies assessing steps per day used pedometers [27,30,39,40], smartphone apps [36,41], telerehabilitation [28,42], and wearable activity trackers in their interventions [35,38]. When examining the effects of each intervention component separately, only smartphone apps (MD 3.07, 95% CI 0.5-5.55) and wearable activity trackers (MD 2.29, 95% CI 1.44-3.13) showed significant improvements in favor of smart technology interventions. Smart technology interventions for studies that assessed total PA included pedometers [43], telerehabilitation [42], websites [45], smartphone apps [36,41], and interventions including multiple smart technology components [33,34,37]. In the subgroup meta-analyses, smartphone apps showed significant improvements in total PA scores compared with face-to-face alone (SMD 0.37, 95% CI 0.10-0.63), and multicomponent interventions favored smart technology, but the effect was not statistically significant (SMD 0.23, 95% CI −0.05 to 0.51).

##### Smart Technology Alone

There was a large variability in the components making up smart technology interventions in the included studies. Only 4 RCTs (n=1701) evaluated the effect of an entirely smart technology alone intervention (ie, did not include any in-person consultations) versus face-to-face alone [28,37,42,45]. Subgroup analyses were performed for both steps per day and total PA scores. For both outcomes, the pooled results were not significant and did not appear to favor either intervention (Figure 2).

**Figure 3:**
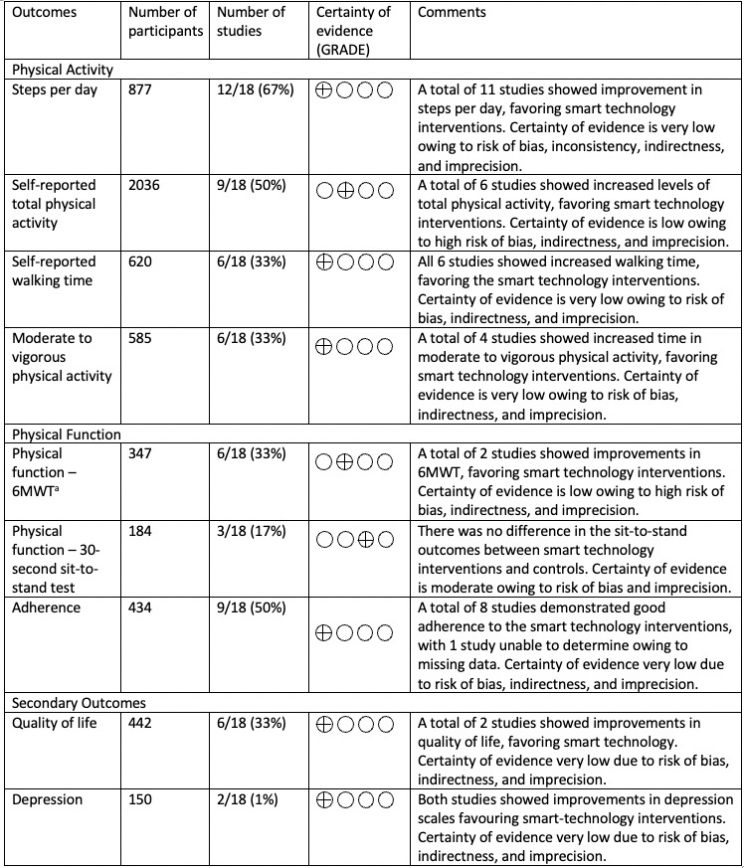
Grading of Recommendations Assessment, Development, and Evaluation (GRADE) summary of findings table.

The evidence for the effect of smart technology interventions on PA was judged to be very low based on the GRADE criteria (Figure 3). Therefore, our confidence in the effect estimate is limited, and the true effect is likely to be different from the estimated effect.

### Physical Function

#### Overview

**Figure 4:**
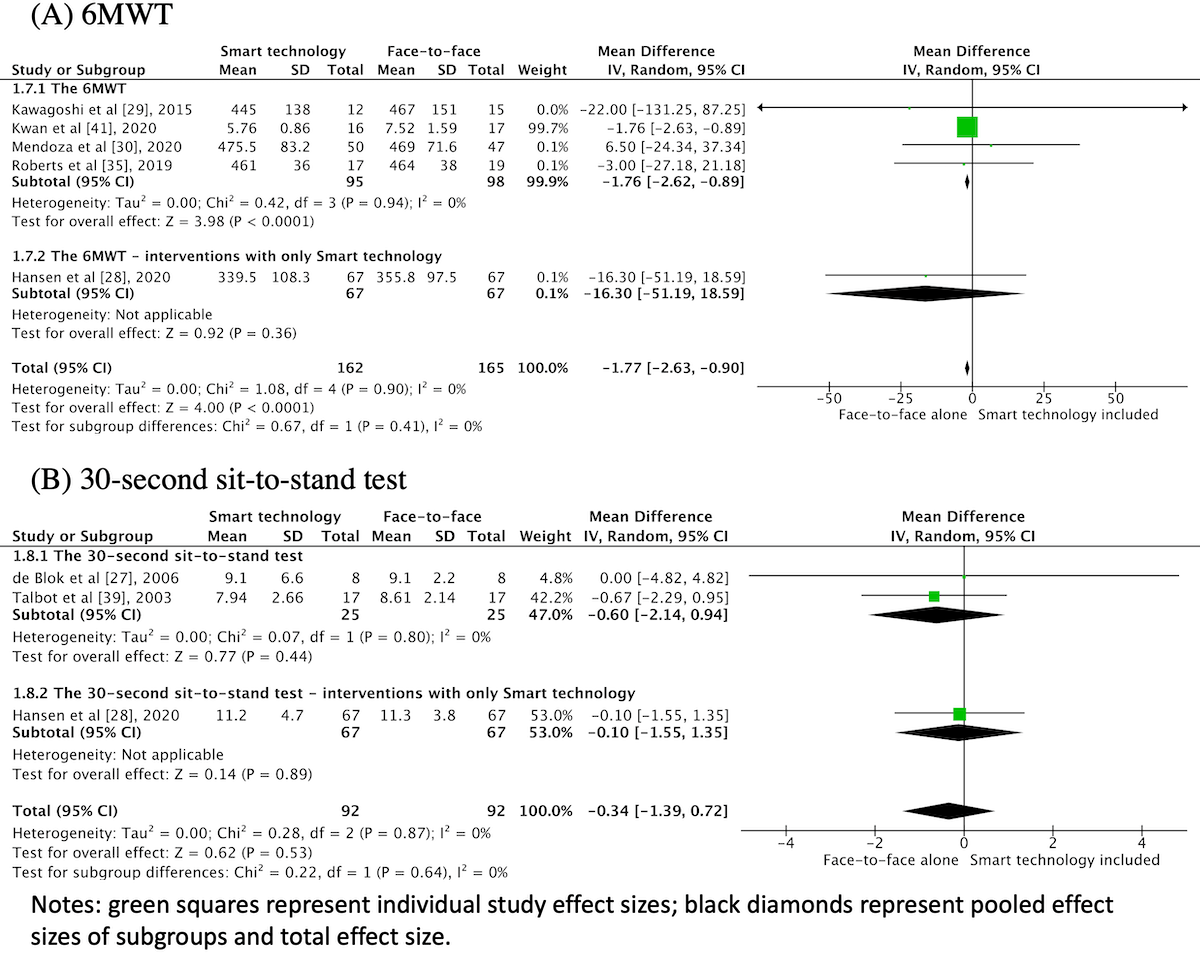
Meta-analysis of smart technology versus face-to-face alone for physical function. 6MWT- 6-minute walk test.

Of 19 studies, 10 (53%) evaluated some aspects of physical function. The most common performance-based measures were the 6-minute walk test (6-MWT) [28-30,35,41] and the 30-second sit-to-stand test [27,28,39]. The remaining 13 measures were only included in 1 or 2 studies (eg, 4-meter gait speed [34], short physical performance battery [34], timed stair climbing [35], peak VO_2_ [14,39], hand grip strength [31,34], maximal inspiratory and expiratory force [29], and quadriceps force) [29,35] and therefore were not pooled (Figure 4). In total, 5 RCTs were pooled in a random effects meta-analysis for 6-MWT including 327 participants, with a MD of −1.77 m (95% CI −2.63 to −0.90) [28,29,32,34,38]. Furthermore, 3 studies (n=184) that used the 30-second sit-to-stand test found no significant difference between interventions (SMD −0.37, 95% CI −1.66 to 0.92). The sensitivity analysis for the 6-MWT test included 3 studies (n=267). After removing those with a high risk of bias, the difference was not statistically significant (MD −3.26, 95% CI −19.97 to 13.45; Multimedia Appendix 1).

#### Subgroup Analyses

##### Smart Technology Intervention Components

We conducted additional meta-analyses on 5 studies that included the 6-MWT as an outcome by type of smart technology intervention component: pedometers [29,30], smartphone apps [41], telerehabilitation [28], and wearable activity trackers [35]. We examined the effects of pedometer interventions on the 6-MWT on their own. There was a nonsignificant MD (4.40, 95% CI −25.28 to 34.07). Smart technology components included in the 3 studies with the 30-second sit-to-stand test were pedometers [27,39] and telerehabilitation [28]. The findings were nonsignificant for pedometers compared with face-to-face alone with an MD of −0.60 (95% CI −2.14 to 0.94). Complete results for these meta-analyses are available in section 6 of Multimedia Appendix 1.

##### Smart Technology Alone

Only 1 study (n=134) examined a smart technology intervention alone using telerehabilitation (video conferencing) [28]. For both the 6-MWT and the 30-second sit-to-stand test, results in this study favored the face-to-face intervention.

The evidence for the effect of smart technology on the 6-MWT and the 30-second sit-to-stand test was judged to be very low based on the GRADE criteria. Therefore, our confidence in the effect estimates is limited, and the true effect may be substantially different from the estimated effect.

### Adherence Rates

A total of 10 studies did not report adherence rates [30,32-34,37,38,40,43,45]. Two studies reported overall adherence to the intervention [28,42], and 7 studies reported adherence to the components of the intervention [27,29,31,35,39,41,44]. Overall, across the available studies, adherence rates to smart technology interventions ranged from 64.8% [42] to 95.5% [27]. Adherence to specific smart technology interventions was reported as follows: pedometers ranged from 76% [39] to 95.5% [27], and telerehabilitation ranged from 64.8% [32] to 85% [28]. The adherence to face-to-face interventions ranged from 64% [28] to 100% [41].

### Secondary Outcomes

Three studies measured depression using different scales, including the Hospital Anxiety and Depression Scale [28], Beck Depression Inventory [27], and Care Depression Instrument [37]. We pooled data from 2 RCTs for a random effects meta-analysis, which showed no significant difference between interventions that included smart technology and face-to-face alone (SMD 0.09, 95% CI −0.23 to 0.41) [27,28]. Furthermore, 6 studies measured HRQoL using the Clinical COPD Questionnaire [28,31], St George’s Respiratory Questionnaire [27,30], HeartQoL global score [33], and Chronic Respiratory Disease Questionnaire [29]. A meta-analysis of 6 studies demonstrated a significant SMD (0.31, 95% CI 0.11-0.51) in favor of smart technology interventions. For HRQoL, we completed a subgroup analysis by smart technology component; multicomponent interventions (n=2; SMD 0.35, 95% CI 0.05-0.66) showed significant improvements compared with face-to-face alone, but pedometers did not (n=3; SMD 0.04, 95% CI −0.49 to 0.57). After performing a sensitivity analysis excluding studies with a high risk of bias, our results did not change the significance or direction of results. Only 1 study examined anxiety using the Hospital Anxiety and Depression Scale and demonstrated an improvement in favor of smart technology at the first follow-up [28]. Meta-analyses for secondary outcomes are available in Multimedia Appendix 1.

## Discussion

### Principal Findings

PA is integral to reducing age-related illness and disability. This review of 19 studies involving 3455 patients found that interventions that include smart technology may improve steps per day, total PA, walking, and HRQoL in older adults. Although the overall quality and certainty of the evidence were judged to be very low and more precise estimates will need to be obtained, our results may have important implications for research and practice on PA promotion, especially in the context of the COVID-19 pandemic and public health restrictions.

Among the included studies, we found that most (n=15) smart technology interventions used multiple components with intervention groups also receiving a face-to-face component. One of the challenges of multicomponent interventions is that it limits our understanding of the effectiveness of smart technology alone. For example, intervention and control groups in several studies received usual care, with the only difference between groups being the addition of a pedometer or wearable activity tracker [29,38-40]. Of the 4 studies examining smart technology alone, 2 examined the effect on steps per day; our subgroup analysis found a nonsignificant difference of 260 steps per day favoring smart technology [28,42]. Notably, the results from these 2 studies were contradictory, with Hansen et al [28] reporting that face-to-face interventions were more effective. However, they evaluated patients with severe COPD and reported a decline in PA for both groups over the course of the study. This may be caused, in part, by the progressive nature of COPD or by the short length of the intervention at only 10 weeks [28]. In line with our meta-analysis for steps per day, the 3 RCTs examining the effect of smart technology alone on total PA showed a small, nonsignificant effect favoring smart technology [37,42,45]. It will be important for future work to determine whether smart technology on its own is more, less, or as effective as face-to-face interventions. This is especially important during the COVID-19 pandemic, as in-person contacts should be limited to minimize the risk of transmission. Conversely, if smart technology interventions alone are less effective, it will be important to revert to including some type of face-to-face component as soon as it is safe and feasible.

Patient-important outcomes were not well represented in our included studies, which highlights an important gap in the design and reporting of smart technology interventions. Among 19 studies, only 7 (37%) unique studies reported on patient-important outcomes: 6 reported on HRQoL [27-31,33], 3 on depression [27,28,37], and 1 on anxiety [28]. Evidence has demonstrated associations between PA levels and mental health and quality of life in older adults [51]. Therefore, it is vital that research includes these measures to further our understanding of the magnitude and direction of these relationships. This is timely as we have seen the prevalence of depression and anxiety increasing over the last 2 years with the pandemic and public health restrictions [52]. Older adults have been shown to be at an increased risk for anxiety [52]. In addition to the lack of patient-important outcomes, we found that data on intervention adherence were largely missing or inconsistently reported. This leads to several challenges, including understanding the acceptability of these interventions for older adults and interpreting results of the primary studies. For example, if adherence to the intervention was poor, it would be difficult to appreciate if the differences (or lack thereof) between groups were because of intervention failure or implementation failure. The issue of acceptability is also crucial, particularly considering that the current literature on the usability of technology in this population is limited.

Our results suggest that the type of smart technology used for PA interventions may influence the effectiveness of the intervention. We found that studies that used smartphone apps led to significant improvements in steps per day and total PA scores [53]. Other systematic reviews examining the effectiveness of mobile phone interventions in adults aged >18 years and >50 years and older adults (>65 years) have shown mixed evidence for improving PA [53-55]. Potential reasons for these discordant results may be the diversity of interventions within control groups, small sample sizes, and moderate to high levels of heterogeneity across studies [53,54]. We also found that wearable activity trackers significantly improved the number of steps per day. Researchers should consider the type of smart technology in conjunction with the PA goals and the population to achieve the best outcome. Further research is warranted to determine the optimal types of smart technology interventions for older adults.

The existing literature has several limitations that warrant future research. Our findings are based on very low quality of evidence, as per the GRADE criteria. All included studies had some concerns or a high risk of bias, most commonly because of missingness of outcomes, lack of intention-to-treat analyses, inadequate allocation concealment, and lack of prespecified statistical analyses or protocols. The variability in populations, smart technology interventions, control group interventions, and outcomes among studies are major contributing factors to the large degree of statistical heterogeneity. Furthermore, most studies used multiple components, making it difficult to assess which parts may have contributed to differences between groups or, conversely, if additive components may have diluted a potentially effective intervention. Importantly, 79% (15/19) of the studies included an element of face-to-face interaction in the intervention group, making it difficult to evaluate the effectiveness of smart technology alone. Due to the limited number of studies and small sample sizes, there is a need to group studies and smart technologies broadly to have sufficient sample sizes to conduct meta-analyses. Given that different smart technologies can be used for different intervention components, it may be difficult to apply our findings in practice when designing interventions. Although we conducted subgroup analyses where there were sufficient data, there is a need for additional research comparing different types of smart technologies for supporting specific PA interventions in older adults [18,19,23]. Finally, our results may have been influenced by the high risk of performance bias caused by the impracticability of blinding therapists and participants owing to the nature of the interventions. Future research should focus on minimizing the risk of bias, evaluating individual smart technology components and smart technology alone, and including standardized control groups. Improved reporting of control group interventions may also assist with interpretation of results.

This review also has some limitations. We did not include gray literature owing to a lack of central sources to identify and retrieve these citations [56]. In addition, we excluded studies published in languages other than English because of the feasibility of the review. Therefore, our cohort of studies may not represent the entirety of the literature. This review has several important strengths. To the best of our knowledge, this is the first study to attempt to compare smart technology interventions specifically with face-to-face interventions, which is critical for determining their effectiveness compared with traditional modes of delivery. In addition, we published our peer-reviewed protocol, and we developed and conducted our search in collaboration with a health research librarian.

### Conclusions

In the context of substantial heterogeneity and very low quality of evidence, our results suggest that PA interventions that include smart technology components may significantly improve steps per day and total PA in community-dwelling older adults. Subgroup analyses showed that smartphone apps and wearable activity trackers seem to be the most effective smart technology, which may be helpful for health care practitioners when determining appropriate methods of remote PA promotion. When comparing smart technology alone with face-to-face alone, there were few studies with discordant results and no significant differences between groups. The results should be interpreted with caution given the challenges with the existing literature cited in the discussion.

## Appendix

Multimedia Appendix 1 Description of data: search strategies, full text exclusion reasons, detailed intervention reasons, risk of bias summary, physical activity meta-analyses, and secondary outcome meta-analyses.

## Abbreviations

COPD: chronic obstructive pulmonary disease
GRADE: Grading of Recommendations Assessment, Development, and Evaluation
HRQoL: health-related quality of life
MD: mean difference
PA: physical activity
PRISMA: Preferred Reporting Items for Systematic Reviews and Meta-Analysis
RCT: randomized controlled trial
SMD: standardized mean difference
