# Supplementary material for: Interventions Including Smart Technology Compared With Face-to-face Physical Activity Interventions in Older Adults: Systematic Review and Meta-analysis
Source: J Med Internet Res. 2022 Oct 31;24(10):e36134. doi: 10.2196/36134 (PMC9664321; doi:10.2196/36134)
Supplement: Multimedia Appendix 1 [file jmir_v24i10e36134_app1.doc]

Supplementary File Table of Contents

[1. SEARCH STRATEGIES 2](#__RefHeading___Toc102648402)

[Table 1. Medline: 4,101 results, February 1, 2021 2](#__RefHeading___Toc102648403)

[Table 2. CINAHL: 1259 results, February 1, 2021 4](#__RefHeading___Toc102648404)

[Table 3. AMED: 212 results, February 1, 2021 6](#__RefHeading___Toc102648405)

[Table 4. Embase: 6673 results, February 1, 2021 8](#__RefHeading___Toc102648406)

[2. FULL TEXT EXCLUSION REASONS 1](#__RefHeading___Toc102648407)

[Table 5. Full text reason for exclusions 1](#__RefHeading___Toc102648408)

[3. Detailed intervention descriptions 30](#__RefHeading___Toc102648409)

[Table 6. Intervention and control group descriptions 30](#__RefHeading___Toc102648410)

[4. RISK OF BIAS SUMMARY 48](#__RefHeading___Toc102648411)

[Figure 1. Risk of bias of all included studies 48](#__RefHeading___Toc102648412)

[5. PHYSICAL ACTIVITY META-ANALYSES 48](#__RefHeading___Toc102648413)

[Figure 2. Steps/day subgroup analyses 48](#__RefHeading___Toc102648414)

[Figure 3. Total PA subgroup analyses 49](#__RefHeading___Toc102648415)

[Figure 4. Sensitivity Analyses 49](#__RefHeading___Toc102648416)

[6. PHYSICAL FUNCTION META-ANALYSES 50](#__RefHeading___Toc102648417)

[Figure 5. The 6MWT subgroup analyses 50](#__RefHeading___Toc102648418)

[Figure 6. The 30-second sit-to-stand subgroup analyses 50](#__RefHeading___Toc102648419)

[Figure 7. Sensitivity analyses 51](#__RefHeading___Toc102648420)

[7. SECONDARY OUTCOME META-ANALYSIS 51](#__RefHeading___Toc102648421)

[Figure 8. Depression meta-analysis 51](#__RefHeading___Toc102648422)

[Figure 9. HRQoL meta-analyses 51](#__RefHeading___Toc102648423)

# 1. SEARCH STRATEGIES

## Table 1. Medline: 4,101 results, February 1, 2021

| 1 | Elder* | | 38 | Increas* |
| --- | --- | --- | --- | --- |
| 2 | Senior | | 39 | Start* |
| 3 | Retire* | | 40 | Adher* |
| 4 | Old* | | 41 | Sustain* |
| 5 | Aged | | 42 | Maintain |
| 6 | Aging | | 43 | Circuit* |
| 7 | Ageing | | 44 | Aqua* |
| 8 | Person | | 45 | Gym* |
| 9 | People | | 46 | Physical activ* |
| 10 | Adult | | 47 | Decreas* |
| 11 | Geriatric* | | 48 | Reduc* |
| 12 | Frail* | | 49 | Discourag* |
| 13 | exp aged/ | | 50 | Sedentary |
| 14 | exp Technology/ | | 51 | Deskbound |
| 15 | exp Telemedicine/ | | 52 | Physical* inactiv* |
| 16 | exp telerehabilitation/ | | 53 | Physical activit* |
| 17 | exp Wearable Electronic Devices/ | | 54 | Physical function |
| 18 | Electronic Mail/ | | 55 | Step count* |
| 19 | exp smartphone/ | | 56 | Pedometer* |
| 20 | exp Videoconferencing/ | | 57 | Sedentary* |
| 21 | Mobile Applications/ | | 58 | Mobilit* |
| 22 | Smart-tech | | 59 | Yoga |
| 23 | Telerehab | | 60 | Walk* |
| 24 | Telemedicine | | 61 | Cycl* |
| 25 | Computer* | | 62 | Bicycl* |
| 26 | Smart watch | | 63 | Swim* |
| 27 | Wearable technology | | 64 | Strength train* |
| 28 | Mobile health | | 65 | Weight lift* |
| 29 | mHealth | | 66 | Skat* |
| 30 | Video call* | | 67 | Jog* |
| 31 | Laptop* | | 68 | Run* |
| 32 | Smart phone* | | 69 | Pilates |
| 33 | Tablet* | | 70 | Leisure activ* |
| 34 | Web-based | | 71 | Hiking |
| 35 | Promot* | | 72 | Sport* |
| 36 | Uptak* | | 73 | Exercis* |
| 37 | Encourag* | | 74 | Physical |
| **Medline continued** | | | | |
| 75 | | Fit* | | |
| 76 | | Train* | | |
| 77 | | Activ* | | |
| 78 | | Endur* | | |
| 79 | | exp exercise | | |
| 80 | | exp physical fitness | | |
| 81 | | exp sports | | |
| 82 | | exp recreation | | |
| 83 | | Running | | |
| 84 | | Swimming | | |
| 85 | | Walking | | |
| 86 | | exp yoga | | |
| 87 | | exp fitness centers | | |
| 88 | | Aerobic capacity | | |
| 89 | | Sedentary behavior | | |
| 90 | | Leisure activities | | |
| 91 | | Leisure | | |
| 92 | | Randomized controlled trial | | |
| 93 | | Controlled clinical trial | | |
| 94 | | Randomized | | |
| 95 | | Placebo | | |
| 96 | | Randomly | | |
| 97 | | Trial | | |
| 98 | | Groups | | |
| 99 | | (2 or 3 or 4 or ((5 or 6 or 7 or 8) adj2 (9 or 10 or 11)) | | |
| 100 | | 1 or 12 or 13 or 99 | | |
| 101 | | 14 or 15 or 16 or 17 or 18 or 19 or 20 or 21 or 22 or 23 or 24 or 25 or 26 or 27 or 28 or 29 or 30 or 31 or 32 or 33 or 34 | | |
| 102 | | (35 or 36 or 37 or 38 or 39 or 40 or 41 or 42) adj5 (43 or 44 or 45 or 46) | | |
| 103 | | (47 or 48 or 49) adj5 (50 or 51 or 52 or 53) | | |
| 104 | | 54 or 55 or 56 or 57 or 58 or 59 or 60 or 61 or 62 or 63 or 64 or 65 or 66 or 67 or 68 or 69 or 70 or 71 or 72 | | |
| 105 | | (73 or 74) adj5 (75 or 76 or 77 or 78) | | |
| 106 | | 79 or 80 or 81 or 82 or 83 or 84 or 85 or 86 or 87 or 88 or 89 or 90 or 91 | | |
| 107 | | 101 or 102 or 103 or 104 or 105 or 106 | | |
| 108 | | 92 or 93 or 94 or 95 or 96 or 97 or 98 | | |
| 109 | | 100 and 107 and 108 | | |

## Table 2. CINAHL: 1259 results, February 1, 2021

| 1 | MH Aged+ | 38 | tablet* |
| --- | --- | --- | --- |
| 2 | Elder* | 39 | web-based |
| 3 | Senior | 40 | mHealth |
| 4 | Retiree | 41 | promot* |
| 5 | Old* | 42 | uptak* |
| 6 | Aged | 43 | Encourag* |
| 7 | Aging | 44 | increas* |
| 8 | Ageing | 45 | start* |
| 9 | Person | 46 | adher* |
| 10 | People | 47 | sustain* |
| 11 | Adult | 48 | maintain* |
| 12 | Geriatric* | 49 | circuit* |
| 13 | Frail* | 50 | aqua* |
| 14 | MH “technology+” | 51 | gym* |
| 15 | MH “Telemedicine+” | 52 | Physical activ* |
| 16 | MH “Telerehabilitation+” | 53 | decreas* |
| 17 | MH “Wearable sensors+” | 54 | reduc* |
| 18 | MH “Email+” | 55 | discourag* |
| 19 | MH “Cellular phone” | 56 | Sedentary |
| 20 | MH “Smartphone+” | 57 | Deskbound |
| 21 | MH “Telehealth+” | 58 | Physical* inactiv* |
| 22 | MH “Teleconferencing+” | 59 | Physical activit* |
| 23 | MH “Videoconferencing+” | 60 | Physical function |
| 24 | MH Mobile “Applications+” | 61 | Step count* |
| 25 | Smart-tech | 62 | pedometer* |
| 26 | Technology | 63 | sedentary* |
| 27 | telerehab* | 64 | mobilit* |
| 28 | Telemedicine | 65 | Yoga |
| 29 | Electronic mail | 66 | walk* |
| 30 | Mobile application* | 67 | cycl* |
| 31 | computer* | 68 | bicycl* |
| 32 | Smart watch | 69 | swim* |
| 33 | Wearable technology | 70 | Strength train* |
| 34 | Mobile health | 71 | Weight lift* |
| 35 | Video call* | 72 | skat* |
| 36 | Laptop* | 73 | jog* |
| 37 | Smart phone* | 74 | run* |

| **CINAHL continued** | | | |
| --- | --- | --- | --- |
| 75 | Pilates | 103 | 25 or 26 or 27 or 28 or 29 or 30 or 31 or 32 or 33 or 34 or 35 or 36 or 37 or 38 or 39 or 40 |
| 76 | Leisure activ* | 104 | (41 or 42 or 43 or 44 or 45 or 46 or 47 or 48) and (49 or 50 or 51 or 52) |
| 77 | Hiking | 105 | (53 or 54 or 55) adj5 (56 or 57 or 58) or (59) |
| 78 | sport* | 106 | 60 or 61 or 62 or 63 or 64 or 65 or 66 or 67 or 68 or 69 or 70 or 71 or 72 or 73 or 74 or 75 or 76 or 77 or 78 |
| 79 | exercis* | 107 | (79 or 80) and (81 or 82 or 83 or 84) |
| 80 | Physical | 108 | 85 or 86 or 87 or 88 or 89 or 90 or 91 or 92 or 93 or 94 or 95 or 96 or 97 |
| 81 | fit* | 109 | 98 or 99 |
| 82 | train* | 110 | 102 or 103 |
| 83 | activ* | 111 | 104 or 105 or 106 or 107 or 108 |
| 84 | endur* | 112 | 101 and 109 and 110 and 111 |
| 85 | MH “exercise” |  |  |
| 86 | MH “physical fitness” |  |  |
| 87 | MH “sports+” |  |  |
| 88 | MH “recreation+” |  |  |
| 89 | MH “running” |  |  |
| 90 | MH “swimming” |  |  |
| 91 | MH “walking” |  |  |
| 92 | MH “yoga+” |  |  |
| 93 | MH “fitness centers+” |  |  |
| 94 | MH “aerobic capacity” |  |  |
| 95 | MH “sedentary behavior” |  |  |
| 96 | MH “leisure activities” |  |  |
| 97 | MH “leisure” |  |  |
| 98 | Randomized control trial |  |  |
| 99 | RCT |  |  |
| 100 | 2 or 3 or 4 or ((5 or 6 or 7 or 8) adj2 (9 or 10 or 11))) |  |  |
| 101 | 1 or 100 or 12 or 13 |  |  |
| 102 | 14 or 15 or 16 or 16 or 17 or 18 or 19 or 20 or 21 or 22 or 23 or 24 |  |  |

## Table 3. AMED: 212 results, February 1, 2021

| 1 | Elder*.mp. | 37 | Sustain*.mp. |
| --- | --- | --- | --- |
| 2 | Senior.mp. | 38 | Maintain*.mp. |
| 3 | Retire*.mp. | 39 | Circuit*.mp. |
| 4 | Old*.mp. | 40 | Aqua*.mp. |
| 5 | Aged.mp. | 41 | Gym*.mp. |
| 6 | Aging.mp. | 42 | Physical activ*.mp. |
| 7 | Ageing.mp. | 43 | Decreas*.mp. |
| 8 | Person.mp. | 44 | Reduc*.mp. |
| 9 | People.mp. | 45 | Discourag*.mp. |
| 10 | Adult.mp. | 46 | Sedentary.mp. |
| 11 | Geriatric* | 47 | Deskbound.mp. |
| 12 | Frail* | 48 | Physical* inactiv*.mp. |
| 13 | exp aged/ | 49 | Physical activit*.mp. |
| 14 | exp Technology/ | 50 | Physical function.mp. |
| 15 | exp Telemedicine/ | 51 | Step count*.mp. |
| 16 | Smart-tech.mp. | 52 | Pedometer*.mp. |
| 17 | Technology.mp. | 53 | Sedentary*.mp. |
| 18 | Telerehab*.mp. | 54 | Mobilit*.mp. |
| 19 | Telemedicine.mp. | 55 | Yoga.mp. |
| 20 | Electronic mail.mp. | 56 | Walk*.mp. |
| 21 | Mobile application*.mp. | 57 | Cycl*.mp. |
| 22 | Computer*.mp. | 58 | Bicycl*.mp. |
| 23 | Smart watch.mp. | 59 | Swim*.mp. |
| 24 | Wearable technology.mp. | 60 | Strength train*.mp. |
| 25 | Mobile health.mp. | 61 | Weight lift*.mp. |
| 26 | mHealth.mp. | 62 | Skat*.mp. |
| 27 | Video call*.mp. | 63 | Jog*.mp. |
| 28 | Laptop*.mp. | 64 | Run*.mp. |
| 29 | Smart phone*.mp. | 65 | Pilates.mp. |
| 30 | Tablet*.mp. | 66 | Leisure activ*.mp. |
| 31 | Promot*.mp. | 67 | Hiking.mp. |
| 32 | Uptak*.mp. | 68 | Sport*.mp. |
| 33 | Encourag*.mp. | 69 | Exercis*.mp. |
| 34 | Increas*.mp. | 70 | Physical.mp. |
| 35 | Start*.mp. | 71 | Fit*.mp. |
| 36 | Adher*.mp. | 72 | Train*.mp. |

| **AMED continued** | |
| --- | --- |
| 73 | Activ*.mp. |
| 74 | Endur*.mp. |
| 75 | exp Exercise |
| 76 | exp Physical fitness |
| 77 | exp Sports |
| 78 | exp Recreation |
| 79 | exp Running |
| 80 | exp Swimming |
| 81 | exp Walking |
| 82 | exp Yoga |
| 83 | exp Sedentary lifestyle |
| 84 | Exp Leisure activities |
| 85 | (1 or 2 or 3 or ((4 or 5 or 6 or 7) adj2 (8 or 9 or 10))).mp. |
| 86 | 11 or 12 or 13 |
| 87 | 85 or 86 |
| 88 | 14 or 15 or 16 or 17 or 18 or 19 or 20 or 21 or 22 or 23 or 24 or 25 or 26 or 27 or 28 or 29 or 30 |
| 89 | ((31 or 32 or 33 or 34 or 35 or 36 or 37 or 38) adj5 (39 or 40 or 41 or 42)).mp. |
| 90 | ((43 or 44 or 45) adj5 (46 or 47 or 48 or 49)).mp. |
| 91 | (50 or 51 or 52 or 53 or 54 or 55 or 56 or 57 or 58 or 59 or 60 or 61 or 62 or 63 or 64 or 65 or 66 or 67 or 68).mp. |
| 92 | ((69 or 70) adj5 (71 or 72 or 73 or 74)).mp. |
| 93 | 75 or 76 or 77 or 78 or 79 or 80 or 81 or 82 or 83 or 84 |
| 94 | 89 or 90 or 91 or 92 or 93 |
| 95 | 87 and 88 and 94 |

## Table 4. Embase: 6673 results, February 1, 2021

| 1 | elder*.mp. | 34 | mHealth.mp. |
| --- | --- | --- | --- |
| 2 | senior.mp. | 35 | video call*.mp. |
| 3 | retire*.mp. | 36 | laptop*.mp. |
| 4 | old*.mp. | 37 | smart phone*.mp. |
| 5 | aged.mp. | 38 | tablet*.mp. |
| 6 | aging.mp. | 39 | web-based.mp. |
| 7 | ageing.mp. | 40 | 24 or 25 or 26 or 27 or 28 or 29 or 30 or 31 or 32 or 33 or 34 or 35 or 36 or 37 or 38 or 39 or 40 |
| 8 | person.mp. | 41 | prompt*.mp. |
| 9 | people.mp. | 42 | uptak*.mp. |
| 10 | adult.mp. | 43 | encourag*.mp. |
| 11 | (1 or 2 or 3 or ((4 or 5 or 6) adj2 (8 or 9 or 10))).mp. | 44 | increase*.mp. |
| 12 | geriatric* | 45 | start*.mp. |
| 13 | frail* | 46 | adher*.mp. |
| 14 | exp Aged/ | 47 | sustain*.mp. |
| 15 | exp Technology/ | 48 | maintain*.mp. |
| 16 | exp Telemedicine/ | 49 | circuit*.mp. |
| 17 | exp Telerehabilitation/ | 50 | aqua*.mp. |
| 18 | exp Wearable Electronic Devices/ | 51 | gym*.mp. |
| 19 | Electronic Mail/ | 52 | physical activ*.mp. |
| 20 | exp Smartphone/ | 53 | ((41 or 42 or 43 or 44 or 45 or 46 or e7 or 48) adj5 (49 or 50 or 51 or 52) |
| 21 | exp Videoconferencing/ | 54 | decreas*.mp |
| 22 | Mobile Applications/ | 55 | reduc*.mp. |
| 23 | 15 or 16 or 17 or 18 or 19 or 20 or 21 or 22 | 56 | discourag*.mp. |
| 24 | smart-tech.mp. | 57 | sedentary.mp. |
| 25 | technology.mp. | 58 | deskbound.mp. |
| 26 | telerehab*.mp. | 59 | physical* inactiv*.mp. |
| 27 | telemedicine.mp. | 60 | physical activit*.mp. |
| 28 | electronic mail.mp. | 61 | ((54 or 55 or 56) adj5 (57 or 58 or 59) or (60)) |
| 29 | mobile application*.mp. | 62 | physical function.mp. |
| 30 | computer*.mp. | 63 | step count*.mp. |
| 31 | smart watch.mp. | 64 | pedometer*.mp. |
| 32 | wearable technology.mp. | 65 | sedentary*.mp. |
| 33 | mobile health.mp. | 66 | mobilit*.mp. |

| **EMBASE continued** | | | |
| --- | --- | --- | --- |
| 67 | yoga.mp. | 94 | Swimming/ |
| 68 | walk*.mp. | 95 | Walking/ |
| 69 | cycl*.mp. | 96 | Exp Yoga/ |
| 70 | bicycl*.mp. | 97 | Exp Fitness centers/ |
| 71 | swim*.mp. | 98 | Aerobic capacity/ |
| 72 | strength train*.mp. | 99 | Sedentary behaviour/ |
| 73 | weight lift*.mp. | 100 | Leisure activities/ |
| 74 | skat*.mp. | 101 | Leisure/ |
| 75 | jog*mp. | 102 | 89 or 90 or 91 or 92 or 93 or 94 or 95 or 96 or 97 or 98 or 99 or 100 or 101 |
| 76 | run*.mp. | 103 | Randomized controlled trial.pt. |
| 77 | pilates.mp. | 104 | Controlled clinical trial.pt. |
| 78 | leisure activ*.mp. | 105 | Randomized.ab. |
| 79 | hiking.mp. | 106 | Placebo.ab. |
| 80 | sport*.mp. | 107 | Randomly.ab. |
| 81 | 62 or 63 or 64 o 65 or 66 or 67 or 68 or 69 or 70 or 71 or 72 or 73 or 74 or 75 or 76 or 77 or 78 or 79 or 80 | 108 | Trial.ab. |
| 82 | exercis*.mp. | 109 | Groups.ab. |
| 83 | physical.mp. | 110 | exp Animals/ |
| 84 | fit*.mp. | 111 | exp Humans/ |
| 85 | train*.mp. | 112 | ((103 or 104) or (105 or 106 or 107 or 108) not (110 or 111) |
| 86 | activ*.mp. | 113 | 11 or 12 or 143 |
| 87 | endur*.mp. | 114 | 23 or 40 |
| 88 | ((82 or 83) adj5 (84 or 85 or 86 or 87) | 115 | 53 or 61 or 81 or 88 or 102 |
| 89 | Exercise/ | 116 | 112 and 113 and 114 and 115 |
| 90 | exp Physical fitness/ |  |  |
| 91 | exp Sports/ |  |  |
| 92 | exp Recreation/ |  |  |
| 93 | Running/ |  |  |

# 2. FULL TEXT EXCLUSION REASONS

## Table 5. Full text reason for exclusions

| **Title** | **Authors** | **Year** | **Exclusion reason** |
| --- | --- | --- | --- |
| 12-months follow-up of pulmonary tele-rehabilitation versus standard pulmonary rehabilitation: A multicenter randomized clinical trial in patients with severe COPD | Godtfredsen N, Frolich A, Bieler T, Beyer N, Kallemose T, Wilcke T, Ostergaard L, Andreassen H.F, Martinez G, Lavesen M, Hansen H. | 2020 | Intervention |
| 2018 John N. Insall Award: Recovery of Knee Flexion with Unsupervised Home Exercise Is Not Inferior to Outpatient Physical Therapy After TKA: A Randomized Trial. | Fleischman AN, Crizer MP, Tarabichi M, Smith S, Rothman RH, Lonner JH, Chen, AF. | 2019 | Intervention |
| Impact of using a pedometer on time spent walking in older adults with type 2 diabetes. | Engel L and Lindner H. | 2006 | Intervention |
| Home-based exercise with telemonitoring guidance in patients with coronary artery disease: Does it improve long-term physical fitness?. | Avila A, Claes J, Buys R, Azzawi M, Vanhees L, Cornelissen V. | 2020 | Intervention |
| Pilot study of a Web-based compliance monitoring device for patients with congestive heart failure. | Artinian NT, Harden JK, Kronenberg MW, Vander Wal JS, Daher E, Stephens Q, Bazzi RI. | 2003 | Intervention |
| Diet or diet plus physical activity versus usual care in patients with newly diagnosed type 2 diabetes: The Early ACTID randomized controlled trial | Andrews R, Cooper A.R, Montgomery A.A, Norcross A.J, Peters T.J, Sharp D.J, Jackson N, Fitzsimons K, Bright J, Coulman K, England C.Y, Gorton J, McLenaghan A, Paxton E, Polet A, Thompson C, Dayan C.M. | 2011 | Intervention |
| A digital health intervention for cardiovascular disease management in primary care (CONNECT) randomized controlled trial | Redfern J, Coorey G, Mulley J, Scaria A, Neubeck L, Hafiz N, Pitt C, Weir K, Forbes J, Parker S, Bampi F, Coenen A, Enright G, Wong A, Nguyen T, Harris M, Zwar N, Chow C.K, Rodgers A, Heeley E, Panaretto K, Lau A, Hayman N, Usherwood T, Peiris D. | 2020 | Intervention |
| Effectiveness of a Blended Physical Therapist Intervention in People with Hip Osteoarthritis, Knee Osteoarthritis, or Both: A Cluster-Randomized Controlled Trial. | Kloek, Corelien JJ, Bossen D, Spreeuwenberg PM, Dekker J, de Bakker DH, Veenhof C. | 2018 | Intervention |
| Outcomes of a multifaceted physical activity regimen as part of a diabetes self-management intervention | King D.K, Estabrooks P.A, Strycker L.A, Toobert D.J, Bull S.S, Glasgow R.E. | 2006 | Intervention |
| Effects of Home-based Telesupervising Rehabilitation on Physical Function for Stroke Survivors with Hemiplegia: A Randomized Controlled Trial | Chen J, Jin W, Dong WS, Jin Y, Qiao FL, Zhou YF, Ren CC. | 2017 | Intervention |
| Home-based telerehabilitation is not inferior to a centre-based program in patients with chronic heart failure: a randomised trial. | Hwang R, Bruning J, Morris NR, Mandrusiak A, Russell T. | 2017 | Intervention |
| Impact of mHealth technology on adherence to healthy PA after stroke: a randomized study. | Grau-Pellicer M, Lalanza JF, Jovell-Fernandez E, Capdevila L. | 2020 | Intervention |
| Feasibility and preliminary effects of a tele-prehabilitation program and an in-person prehablitation program compared to usual care for total hip or knee arthroplasty candidates: a pilot randomized controlled trial. | Doiron-Cadrin P, Kairy D, Vendittoli P-A, Lowry V, Poitras S, Desmeules F. | 2020 | Intervention |
| Effects of a 12-Week mHealth Program on Functional Capacity and Physical Activity in Patients with Peripheral Artery Disease. | Duscha BD, Piner LW, Patel MP, Crawford LE, Jones WS, Patel MR, Kraus WE. | 2018 | Intervention |
| Telehealth for patients at high risk of cardiovascular disease: pragmatic randomised controlled trial. | Salisbury C, O'Cathain A, Thomas C, Edwards L, Gaunt D, Dixon P, Hollinghurst S, Nicholl J, Large S, Yardley L, Fahey T, Foster A, Garner K, Horspool K, Man M-S, Rogers A, Pope C, Montgomery, Alan A. | 2016 | Intervention |
| A comparison of televideo and traditional in-home rehabilitation in mobility impaired older adults. | Sanford JA, Hoenig H, Griffiths PC, Butterfield T, Richardson P, Hargraves K. | 2007 | Intervention |
| Impact of novel smartphone application on pain and mobility in osteoarthritis patients treated with hylan g-f 20 | Skrepnik N, Spitzer A, Altman R, Hoekstra J.A, Stewart J, Toselli R. | 2016 | Intervention |
| Technology-Assisted Balance and Gait Training Reduces Falls in Patients with Parkinson’s Disease: A Randomized Controlled Trial With 12-Month Follow-up. | Shen X and Mak MKY. | 2015 | Intervention |
| Augmented community telerehabilitation intervention to improve outcomes for people with stroke AKTIV-a randomised controlled trial | Say Well N, Vandal A.C, Taylor D. | 2017 | Intervention |
| Beneficial effect of personalized lifestyle advice compared to generic advice on wellbeing among Dutch seniors - An explorative study. | Doets EL, de Hoogh IM, Holthuysen N, Wopereis S, Verain MCD, van den Puttelaar J, Hogenelst K, Boorsma A, Bouwman EP, Timmer M, Pasman WJ, van Erk M, Reinders MJ. | 2019 | Intervention |
| Effectiveness of an interactive virtual telerehabilitation system in patients after total knee arthroplasty: a randomized controlled trial. | Piqueras M, Marco E, Coll M, Escalada F, Ballester A, Cinca C, Belmonte RM, Josep M. | 2013 | Intervention |
| Wearable Sensor Technology Efficacy in Peripheral Vascular Disease (wSTEP): A Randomized Controlled Trial. | Normahani P, Kwasnicki R, Bicknell C, Allen L, Jenkins MP, Gibbs R, Cheshire N, Darzi A, Riga C. | 2018 | Intervention |
| A telehealth program for self-management of COPD exacerbations and promotion of an active lifestyle: a pilot randomized controlled trial. | Tabak M, Brusse-Keizer M, van der Valk P, Hermens H, Vollenbroek-Hutten M. | 2014 | Intervention |
| Telerehabilitation is non-inferior to usual care following total hip replacement -- a randomized controlled non-inferiority trial. | Nelson M, Bourke M, Crossley K, Russell T. | 2020 | Intervention |
| Patient Satisfaction with In-Home Telerehabilitation After Total Knee Arthroplasty: Results from a Randomized Controlled Trial. | Moffet H, Tousignant M, Nadeau S, Merette C, Boissy P, Corriveau H, Marquis F, Cabana F, Belzile EL, Ranger P, Dimentberg R. | 2017 | Intervention |
| A randomized controlled trial of home telerehabilitation for post-knee arthroplasty. | Tousignant, Michel; Moffet, Helene; Boissy, Patrick; Corriveau, Helene; Cabana, Francois; Marquis, Francois | 2011 | Intervention |
| In-home telerehabilitation compared with face to-face rehabilitation after total knee arthroplasty: A noninferiority randomized controlled trial | Moffet H, Tousignant M, Nadeau S, Merette C, Boissy P, Corriveau H, Marquis F, Cabana F, Ranger P, Belzile E.L, Dimentberg R. | 2015 | Intervention |
| Efficacy of an mHealth intervention to stimulate physical activity in COPD patients after pulmonary rehabilitation. | Vorrink SNW, Kort HSM, Troosters T, Zanen P, Lammers J-WJ. | 2016 | Intervention |
| Effects of an animated diagram and video-based online breathing program for dyspnea in patients with stable COPD | Liu F, Cai H, Tang Q, Zou Y, Wang H, Xu Z, Wei Z, Wang W, Cui J. | 2013 | Intervention |
| Comparison of telecommunication, community, and home-based Tai Chi exercise programs on compliance and effectiveness in elders at risk for falls | Wu G, Keyes L, Callas P, Ren X, Bookchin B. | 2010 | Intervention |
| Benefits and costs of home pedometer assisted physical activity in patients with COPD. A preliminary randomized controlled trial | Widyastuti K, Makhabah D.N, Setijadi A.R, Sutanto Y.S, Suradi, Ambrosino N. | 2018 | Intervention |
| A mobile health application to support self-management in patients with chronic obstructive pulmonary disease: a randomised controlled trial. | Wang LH, Guo YM, Wang M, Zhao Y. | 2021 | Intervention |
| Mobile-phone-based home exercise training program decreases systemic inflammation in COPD: a pilot study. | Wang H-H, Chou P-C, Joa W-C, Chen L-F, Sheng T-F, Ho S-C, Lin H-C, Huang C-D, Chung F-T, Chung KF, Kuo H-F. | 2014 | Intervention |
| Evaluating a Web-Based Coaching Program Using Electronic Health Records for Patients with Chronic Obstructive Pulmonary Disease in China: Randomized Controlled Trial. | Wang L, He L, Tao Y, Sun L, Zheng H, Zheng Y, Shen Y, Liu S, Zhao Y, Wang Y. | 2017 | Intervention |
| A web-based program improves physical activity outcomes in a primary care angina population: randomized controlled trial. | Devi R, Powell J, Singh S. | 2014 | Comparator |
| Feasibility, Acceptability, and Behavioral Outcomes from a Technology-enhanced Behavioral Change Intervention (Prostate 8): A Pilot Randomized Controlled Trial in Men with Prostate Cancer. | Kenfield SA, Van Blarigan EL, Ameli N, Lavaki E, Cedars B, Paciorek AT, Monroy C, Tantum LK, Newton RU, Signorell C, Suh JH, Zhang L, Cooperberg MR, Carroll PR, Chan JM. | 2019 | Comparator |
| Short-term efficacy of a computer-tailored physical activity intervention for prostate and colorectal cancer patients and survivors: a randomized controlled trial. | Golsteijn RHJ, Bolman C, Volders E, Peels DA, de Vries H, Lechner, L. | 2018 | Comparator |
| Feasibility, Acceptability, and Clinical Effectiveness of a Technology-Enabled Cardiac Rehabilitation Platform (Physical Activity Toward Health-I): Randomized Controlled Trial. | Claes J, Cornelissen V, McDermott C, Moyna N, Pattyn N, Cornelis N, Gallagher A, McCormack C, Newton H, Gillain A, Budts W, Goetschalckx K, Woods C, Moran K, Buys R. | 2020 | Intervention |
| Primary care pedometer-based walking intervention: Mixed-methods results from 3-year follow-up of PACE-UP cluster-randomised controlled trial | Wahlich C, Cook D, Kerry S, Limb E, Victor C, Iliffe S, Ussher M, Whincup P, Ekelund U, Fox-Rushby J, Furness C, Beighton C, Normansell R, Ibison J, DeWilde S, Harris T. | 2017 | Conference study |
| Results from a multi-site web-based physical activity intervention in COPD: Between group and site differences | Robinson S.A, Goldstein R.L, Cruz Rivera P.N, Kadri R, Cooper J.D, Richardson C.R, Moy M.L. | 2020 | Conference study |
| Exercise Interventions with Trained Home Helpers for Preventing Loss of Autonomy and Falls in Community-Dwelling Older Adults Receiving Home Heath Physical Therapy T4H: A Randomized Controlled Pilot Study. | Meziere A, Oubaya N, Michel-Pellegrino V, Boudin B, Neau M, Robert H, Cara I, Salgado Sanchez L, Baloul S, Piette F, Pautas E, Picou Y, Curtis V, Schonheit C, Canoui-Poitrine F, Moreau C. | 2021 | Intervention |
| Personalized e-coaching in cardiovascular risk reduction: A randomized controlled trial | Khanji M.Y, Balawon A, Boubertakh R, Hofstra L, Narula J, Hunink M, Pugliese F, Petersen S.E. | 2019 | Study design |
| Pulmonary tele-rehabilitation versus conventional pulmonary rehabilitation a multicenter, single blinded, superiority rct | Hansen H, Frolich A, Beyer N, Bieler T, Kallemose T, Godtfredsen N. | 2019 | Conference study |
| Patient-reported outcomes of usability and enjoyment of using digital devices in rehabilitation as part of the AMOUNT (Activity and Mobility Using Technology) randomised controlled trial | Hassett L, Van Den Berg M, Weber H, Chagpar S, Wong S, McCluskey A, Schurr K, Crotty M, Sherrington C. | 2019 | Conference study |
| The short-term and long-term cost-effectiveness of a pedometer-based intervention in primary care: A within trial analysis and beyond-trial modelling | Anokye N, Fox-Rushby J, Sanghera S, Cook D.G, Kerry S.M, Limb E, Victor C.R, Iliffe S, Shah S.M, Ussher M, Whincup P.H, Ekelund U, Furness C, Ibison J, DeWilde S, David L, Howard E, Dale R, Smith J, Harris T.J. | 2016 | Conference study |
| Motivational Interviewing to Increase Physical Activity in Persons with Parkinson's Disease | Ehrlich-Jones L, Engel E, Song J, Mann K, Greene A, Pedersen J.P, Lee J, Bega D. | 2019 | Conference study |
| Effect of home pulmonary rehab plus health coaching on COPD self-management: A randomized study | Benzo R, Hoult J.P, Thomas B.E, Lam N, Seifert S, Kramer K. | 2019 | Conference study |
| Web/Internet-based telemonitoring of a randomised controlled trial evaluating the time-integrated effects of a 24-week multicomponent intervention on key health outcomes in patients with fibromyalgia | Salaffi F, Ciapetti A, Gasparini S, Atzeni F, Sarzi-Puttini P, Baroni M. | 2015 | Mean age < 60 |
| Feasibility and effects of home-based smartphone-delivered automated feedback training for gait in people with Parkinson's disease: A pilot randomized controlled trial. | Ginis P, Nieuwboer A, Dorfman M, Ferrari A, Gazit E, Canning CG, Rocchi L, Chiari L, Hausdorff JM, Mirelman A. | 2016 | Intervention |
| Effects of a short message service-guided training after acute stroke or TIA (STROKEWALK): A randomized controlled trial | Lundstrom E, Eriksson S, Holmback U, Cederholm T, Vahlberg B. | 2019 | Conference study |
| Providing heart failure rehabilitation in the home via telerehabilitation | Hwang R, Bruning J, Mandrusiak A, Morris N, Russell T. | 2016 | Conference study |
| Efficacy of a computer-based intervention to promote walking in older adults | Paasche-Orlow M, Silliman R, Winter M, Cheng D, Henault L, Bickmore T. | 2012 | Conference study |
| 1-year follow-up of pulmonary tele-rehabilitation versus conventional pulmonary rehabilitation: A multicenter, single blinded, superiority RCT | Hansen H, Bieler T, Beyer N, Kallemose T, Frolich A, Godtfredsen N. | 2019 | Conference study |
| A digital health intervention to improve physical activity after cardiac rehabilitation: The mobile4heart clinical trial | Park L.G, Elnaggar A, Merek S, McCulloch C.E, Whooley M.A. | 2019 | Conference study |
| Research and effect evaluation of community continuous rehabilitation exercise training technology for elderly osteoporosis | Sun C, Xiao Z, Yang P, Huang J, Liu Y, Zhang F, Zhang X, Xi Z, Guo M, Jiang X. | 2020 | Conference study |
| Is Virtual Autonomous Physiotherapist Tele-Rehabilitation Program in Chronic Obstructive Pulmonary Disease equal to Hospital-Based Pulmonary Rehabilitation? | De Las Heras J.C, Balbino F, Hilberg O, Lokke A, Bendstrup E. | 2020 | Conference study |
| A European randomised controlled trial for m-health guided cardiac rehabilitation in the elderly; results of the EU-CaRE RCT study | De Kluiver E.P, Van Der Velde A.E, Meindersma E.P, Prins L.F, Wilhelm M, Iliou M.C, Pena Gil C, Gonzalez-Juanatey J.R, Snoek J.A, Kolkman E, Van't Hof A.W.J, Prescott E. | 2019 | Conference study |
| Multidisciplinary telehealth program for patients affected by chronic heart failure and chronic obstructive pulmonary disease | Scalvini S, Bernocchi P, Baratti D, Gatti T, Paneroni M, La Rovere M.T, Volterrani M, Vitacca M. | 2016 | Conference study |
| Does a Preventive Telemonitoring Service improve the quality of life of frail elderly? Preliminary results of the European project Dreaming | Coll J, Sanjoaquin A.C, Lopez M, Romero D, Pinilla R, Ochoa P. | 2011 | Conference study |
| Preliminary physical activity findings from a home-based Physical Activity and Nutrition Program for Seniors (PANS) | Burke L, Jancey J, Howat P, Lee A, Kerr D, Shilton T, Hills A, Anderson A. | 2011 | Conference study |
| Is predictive information together with adequate physical activity more effective than educational advice on BMI levels in elderly people? | Akira K and Masahiro N. | 2010 | Conference study |
| Telecoaching programme for type 2 diabetes control: a randomised clinical trial. | de Vasconcelos HCA, Lira Neto JCG, de Araujo MRM, Carvalho GCN, de Souza Teixeira CR, de Freitas RWJF, Damasceno MMC. | 2018 | Intervention |
| Cluster randomized controlled trial of a multilevel physical activity intervention for older adults | Kerr J, Rosenberg D, Millstein R.A, Bolling K, Crist K, Takemoto M, Godbole S, Moran K, Natarajan L, Castro-Sweet C, Buchner D. | 2018 | Setting |
| Digital Monitoring of Sleep, Meals, and Physical Activity for Reducing Depression in Older Spousally-Bereaved Adults: A Pilot Randomized Controlled Trial. | Stahl ST, Smagula SF, Dew MA, Schulz R, Albert SM, Reynolds, CF 3rd | 2020 | Outcomes |
| Randomized controlled pilot study of an educational video plus telecare for the early outpatient management of musculoskeletal pain among older emergency department patients. | Platts-Mills TF, Hollowell AG, Burke GF, Zimmerman S, Dayaa JA, Quigley BR, Bush M, Weinberger M, Weaver MA. | 2018 | Intervention |
| Impact of mobile phone text messaging intervention on adherence among patients with diabetes in a rural setting: A randomized controlled trial. | Owolabi EO, Goon DT, Ajayi AI. | 2020 | Outcomes |
| Randomized controlled trial of an internet-based versus face-to-face dyspnea self-management program for patients with chronic obstructive pulmonary disease: pilot study. | Nguyen HQ, Donesky-Cuenco DA, Wolpin S, Reinke LF, Benditt JO, Paul SM, Carrieri-Kohlman V. | 2008 | Comparator |
| Do pedometers increase physical activity in sedentary older women? A randomized controlled trial. | McMurdo MET, Sugden J, Argo I, Boyle P, Johnston DW, Sniehotta FF, Donnan PT | 2010 | Comparator |
| Strength-balance supplemented with computerized cognitive training to improve dual task gait and divided attention in older adults: a multicenter randomized-controlled trial. | van Het Reve E and de Bruin ED. | 2014 | Setting |
| A 2-year follow-up of a lifestyle physical activity versus a structured exercise intervention in older adults | Opdenacker J, Delecluse C, Boen F | 2011 | Intervention |
| Compliance and satisfaction with home exercise: A comparison of computer-assisted video instruction and routine rehabilitation practice | Lysack C, Dama M, Neufeld S, Andreassi E. | 2005 | Setting |
| HIP Mobile: A community-based monitoring, rehabilitation and learning e-system for patients following a hip fracture | Abou-Sharkh A, Mayo N.E, Wall M, Albers A, Bergeron S, Jean S, Berube P, Harvey E.J, Morin S.N. | 2018 | Conference study |
| Home-based telerehabilitation via real-time videoconferencing improves endurance exercise capacity in patients with COPD: The randomized controlled TeleR Study. | Tsai LLY, McNamara RJ, Moddel C, Alison JA, McKenzie DK, McKeough ZJ. | 2017 | Comparator |
| Randomised controlled trial to evaluate the efficacy and usability of a computerised phone-based lifestyle coaching system for primary and secondary prevention of stroke. | Spassova L, Vittore D, Droste DW, Rösch N. | 2016 | Comparator |
| Effectiveness of sensor monitoring in a rehabilitation program for older patients after hip fracture: A three-arm stepped wedge randomized trial | Pol M, Ter Riet G, Van Harting-Sveldt M, Krose B, Buurman B.M. | 2019 | Setting |
| Home-based telemonitored Nordic walking training is well accepted, safe, effective and has high adherence among heart failure patients, including those with cardiovascular implantable electronic devices: a randomised controlled study. | Piotrowicz E, Zielinski T, Bodalski R, Rywik T, Dobraszkiewicz-Wasilewska B, Sobieszczanska-Malek M, Stepnowska M, Przybylski A, Browarek A, Szumowski L, Piotrowski W, Piotrowicz R. | 2015 | Comparator |
| Impact of customized videotape education on quality of life in patients with chronic obstructive pulmonary disease. | Petty TL, Dempsey EC, Collins T, Pluss W, Lipkus I, Cutter GR, Chalmers R, Mitchell A, Weil KC. | 2006 | Intervention |
| Home-based telehealth exercise training program in Chinese patients with heart failure: A randomized controlled trial. | Peng X, Su Y, Hu Z, Sun X, Li X, Dolansky MA, Qu M, Hu X. | 2018 | Comparator |
| Pedometer step count targets during pulmonary rehabilitation in chronic obstructive pulmonary disease: A randomized controlled trial | Nolan C.M, Maddocks M, Canavan J.L, Jones S.E, Delogu V, Kaliaraju D, Banya W, Kon S.S.C, Polkey M.I, Man W.D.-C. | 2017 | Comparator |
| Effect of Remote Monitoring on Discharge to Home, Return to Activity, and Rehospitalization After Hip and Knee Arthroplasty: A Randomized Clinical Trial. | Mehta SJ, Hume E, Troxel AB, Reitz C, Norton L, Lacko H, McDonald C, Freeman J, Marcus N, Volpp KG, Asch DA. | 2020 | Setting |
| Effects of Lifestyle Physical Activity on Vascular Function in Asymptomatic Peripheral Arterial Disease. | Laslovich S, Alvar BA, Allison M, Rauh MJ | 2020 | Comparator |
| Effects of home-based training with telemonitoring guidance in low to moderate risk patients entering cardiac rehabilitation: short-term results of the FIT@Home study. | Kraal JJ, Peek N, Van den Akker-Van Marle, ME, Kemps HM. | 2014 | Intervention |
| Long-term effects of pedometer-based physical activity coaching in severe COPD: A randomized controlled trial | Kohlbrenner D, Sievi N.A, Senn O, Kohler M, Clarenbach C.F. | 2020 | Intervention |
| Effects of low-intensity exercise and home-based pulmonary rehabilitation with pedometer feedback on physical activity in elderly patients with chronic obstructive pulmonary disease | Kawagoshi A, Kiyokawa N, Sugawara K, Takahashi H, Sakata S, Satake M, Shioya T. | 2015 | Duplicate |
| A Primary Care Nurse-Delivered Walking Intervention in Older Adults: PACE (Pedometer Accelerometer Consultation Evaluation)-Lift Cluster Randomised Controlled Trial | Harris T, Kerry S.M, Victor C.R, Ekelund U, Woodcock A, Iliffe S, Whincup P.H, Beighton C, Ussher M, Limb E.S, David L, Brewin D, Adams F, Rogers A, Cook D.G. | 2015 | Intervention |
| Effectiveness of a community- based exercise training programme to increase physical activity level in patients with chronic obstructive pulmonary disease: A randomized controlled trial. | Varas AB, Cordoba S, Rodriguez-Andonaegui I, Rueda, MR, Garcia-Juez S, Vilaro J. | 2018 | Comparator |
| Effect of a Primary Care Walking Intervention with and without Nurse Support on Physical Activity Levels in 45- to 75-Year-Olds: The Pedometer and Consultation Evaluation (PACE-UP) Cluster Randomised Clinical Trial | Harris T, Kerry S.M, Limb E.S, Victor C.R, Iliffe S, Ussher M, Whincup P.H, Ekelund U, Fox-Rushby J, Furness C, Anokye N, Ibison J, DeWilde S, David L, Howard E, Dale R, Smith J, Cook D.G. | 2017 | Comparator |
| Efficacy of a computerized simulation in promoting walking in individuals with diabetes. | Gibson B, Marcus RL, Staggers N, Jones J, Samore M, Weir C. | 2012 | Comparator |
| Wristband accelerometers to motivate arm exercise after stroke (WAVES): A pilot randomized controlled trial. | Da Silva RH, Moore SA, Van Wijck F, Shaw RH, Jackson D, Balaam M, Sutcliffe L, Brkic L, Ploetz T, Price C. I. | 2018 | Setting |
| Feasibility and preliminary efficacy of a telehealth approach to group tango instruction for people with Parkinson's disease | Seidler K.J, Duncan R.P, McNeely M.E, Hackney M.E, Earhart G.M. | 2016 | Study design |
| Can the COPD web be used to promote self-management in patients with COPD in Swedish primary care: a controlled pragmatic pilot trial with 3 month- and 12-month follow-up. | Nyberg A, Tistad M, Wadell K. | 2019 | Study design |
| Pedometer-facilitated walking intervention shows promising effectiveness for reducing cancer fatigue: a pilot randomized trial. | Mayo NE, Moriello C, Scott SC, Dawes D, Auais M, Chasen M. | 2014 | Mean age < 60 |
| Smartphone-based visual feedback trunk control training for gait ability in stroke patients: A single-blind randomized controlled trial. | Shin DC. | 2020 | Mean age < 60 |
| Effect of novel technology-enabled multidimensional physical activity feedback in primary care patients at risk of chronic disease-- the MIPACT study: a randomised controlled trial. | Peacock OJ, Western MJ, Batterham AM, Chowdhury EA, Stathi A, Standage M, Tapp A, Bennett P, Thompson D. | 2020 | Comparator |
| Randomized trial of an internet-based computer-tailored expert system for physical activity in patients with heart disease. | Reid RD, Morrin LI, Beaton LJ, Papadakis S, Kocourek J, McDonnell L, Slovinec D’Angelo ME, Tulloch H, Suskin N, Unsworth K, Blanchard C, Pipe AL. | 2012 | Mean age < 60 |
| Pilot Randomised Controlled Trial of a Web-Based Intervention to Promote Healthy Eating, Physical Activity and Meaningful Social Connections Compared with Usual Care Control in People of Retirement Age Recruited from Workplaces. | Lara J, O'Brien N, Godfrey A, Heaven B, Evans EH, Lloyd S, Moffatt S, Moynihan PJ, Meyer TD, Rochester L, Sniehotta FF, White M, Mathers JC. | 2016 | Comparator |
| Self-tracking of Physical Activity in People with Type 2 Diabetes: A Randomized Controlled Trial. | Kooiman TJM, de Groot M, Hoogenberg K, Krijnen WP, van der Schans CP, Kooy A. | 2018 | Mean age < 60 |
| Use of currently available smart home technology by frail elders: process and outcomes. | Tomita MR, Mann WC, Stanton K, Tomita AD, Sundar V. | 2007 | Intervention |
| A Pilot Randomized Controlled Trial Using SystemCHANGE^TM^ Approach to Increase Physical Activity in Older Kidney Transplant Recipients | O'Brien T, Russell C.L, Tan A, Mion L, Rose K, Focht B, Daloul R, Hathaway D. | 2020 | Comparator |
| Therapeutic benefit of preventive telehealth counseling in the Community Outreach Heart Health and Risk Reduction Trial. | Nolan RP, Upshur REG, Lynn H, Crichton T, Rukholm E, Stewart DE, Alter DA, Chessex C, Harvey PJ, Grace SL, Picard L, Michel I, Angus J, Corace K, Barry-Bianchi SM, Chen MH. | 2011 | Mean age < 60 |
| The impact of different degrees of feedback on physical activity levels: A 4-week intervention study | Van Hoye K, Boen F, Lefevre J. | 2015 | Mean age < 60 |
| Efficacy of an Individually Tailored, Internet-Mediated Physical Activity Intervention in Older Adults: A Randomized Controlled Trial. | Rowley TW, Lenz EK, Swartz AM, Miller NE, Maeda H, Strath SJ. | 2019 | Comparator |
| Effect of a pedometer based aerobic walking program on pain and function among elderly patients with knee osteoarthritis | Shahine N.F, El Ashri N.I, Senna M.K, Abd Elhameed S.H. | 2020 | Comparator |
| Long-term integrated telerehabilitation of COPD Patients: a multicenter randomised controlled trial (iTrain). | Zanaboni P, Dinesen B, Hjalmarsen A, Hoaas H, Holland AE, Carneiro Oliveira C, Wootton R, Oliveira CC. | 2016 | Study design |
| A comparison of the impact of physical exercise, cognitive training and combined intervention on spontaneous walking speed in older adults. | Pothier K, Gagnon C, Fraser SA, Lussier M, Desjardins-Crepeau L, Berryman N, Kergoat M-J, Vu TTM, Li KZH, Bosquet L, Bherer L. | 2018 | Intervention |
| Development of a novel mind-body activity and pain management program for older adults with cognitive decline | Mace R.A, Gates M.V, Bullard B, Lester E.G, Silverman I.H, Quiroz Y.T, Vranceanu A.-M. | 2020 | Study design |
| Effects of Activity Tracker Use with Health Professional Support or Telephone Counseling on Maintenance of Physical Activity and Health Outcomes in Older Adults: Randomized Controlled Trial | Brickwood K.-J, Ahuja K.D.K, Watson G, O'Brien J.A, Williams A.D. | 2021 | Comparator |
| Effectiveness of User- and Expert-Driven Web-based Hypertension Programs: an RCT. | Liu S, Brooks D, Thomas SG, Eysenbach G, Nolan RP. | 2018 | Mean age < 60 |
| Cognitive and physical rehabilitation of intensive care unit survivors: results of the RETURN randomized controlled pilot investigation. | Jackson JC, Ely EW, Morey MC, Anderson VM, Denne LB, Clune J, Siebert CS, Archer KR, Torres R, Janz D, Schiro E, Jones J, Shintani AK, Levine B, Pun BT, Thompson J, Brummel NE, Hoenig H. | 2012 | Mean age < 60 |
| Effectiveness of a video-based exercise programme to reduce falls and improve health-related quality of life among older adults discharged from hospital: a pilot randomized controlled trial. | Haines TP, Russell T, Brauer SG, Erwin S, Lane P, Urry S, Jasiewicz J, Condie P. | 2009 | Intervention |
| Telehealth Versus In-Person Acceptance and Commitment Therapy for Chronic Pain: A Randomized Noninferiority Trial. | Herbert MS, Afari N, Liu L, Heppner P, Rutledge T, Williams K, Eraly S, VanBuskirk K, Nguyen C, Bondi M, Atkinson JH, Golshan S, Wetherell JL. | 2017 | Mean age < 60 |
| Effect of progressive pedometer-based walking intervention on quality of life and general well-being among patients with type 2 diabetes | Guglani R, Shenoy S, Sandhu J.S. | 2014 | Mean age < 60 |
| Effects of tailored telemonitoring on functional status and health-related quality of life in patients with heart failure | Gingele A.J, Ramaekers B, Brunner-La Rocca H.P, De Weerd G, Kragten J, van Empel V, van der Weg K, Vrijhoef H.J.M, Gorgels A, Cleuren G, Boyne J.J.J, Knackstedt C. | 2019 | Study design |
| Promoting physical activity in geriatric patients with cognitive impairment after discharge from ward-rehabilitation: a feasibility study. | Eckert T, Bongartz M, Ullrich P, Abel B, Christian W, Kiss R, Hauer K. | 2020 | Comparator |
| Enhancing physical activity in cardiac patients who report hopelessness: Feasibility testing of an intervention. | Dunn SL, Robbins LB, Smith SW, Ranganathan R, DeVon HA, Collins EG, Hong HG, Tintle NL. | 2019 | Mean age < 60 |
| Tele-rehabilitation program in idiopathic pulmonary fibrosis | De Las Heras J.C, Hilberg O, Lokke A, Bendstrup E. | 2019 | Intervention |
| A Home- and Community-Based Physical Activity Program Can Improve the Cardiorespiratory Fitness and Walking Capacity of Stroke Survivors. | Marsden DL, Dunn A, Callister R, McElduff P, Levi CR, Spratt NJ. | 2016 | Study design |
| Effects of a 12-week mHealth program on peak VO2 and physical activity patterns after completing cardiac rehabilitation: A randomized controlled trial. | Duscha BD, Piner LW, Patel MP, Craig KP, Brady M, McGarrah RW 3rd, Chen C, Kraus WE. | 2018 | Comparator |
| A Comprehensive Intervention for Promoting Successful Aging Amongst Older People with Diabetes with Below-Normal Cognitive Function-A Feasibility Study. | Natovich R, Gayus N, Azmon M, Michal H, Twito OG, Yair T, Raudoi S, Kapra O, Cukierman-Yaffe T. | 2020 | Study design |
| Acceptability and Effects of Commercially Available Activity Trackers for Chronic Pain Management Among Older African American Adults. | Janevic MR, Shute V, Murphy SL, Piette JD. | 2020 | Comparator |
| Web-based intervention to promote physical activity by sedentary older adults: randomized controlled trial. | Irvine AB, Gelatt VA, Seeley JR, Macfarlane P, Gau JM. | 2013 | Comparator |
| Effect of telerehabilitation on mobility in people after hip surgery: a pilot feasibility study. | Kalron A, Tawil H, Peleg-Shani S, Vatine JJ. | 2018 | Comparator |
| Real-time telehealth for COPD self-management using Skype^TM^. | Nield M and Soo Hoo GW. | 2012 | Intervention |
| Effects of multidisciplinary internet-based program on management of heart failure | Tomita M.R, Tsai B.-M, Fisher N.M, Kumar N.A, Wilding G, Stanton K, Naughton B.J. | 2009 | Comparator |
| A pedometer-based walking intervention in 45- to 75-year-olds, with and without practice nurse support: the PACE-UP three-arm cluster RCT. | Harris T, Kerry S, Victor C, Iliffe S, Ussher M, Fox-Rushby J, Whincup P, Ekelund U, Furness C, Limb E, Anokye N, Ibison J, DeWilde S, David L, Howard E, Dale R, Smith J, Normansell R, Beighton C, Morgan K, Wahlich C, Sanghera S, Cook D. | 2018 | Comparator |
| The Home-Heart-Walk study, a self-administered walk test on perceived physical functioning, and self-care behaviour in people with stable chronic heart failure: A randomized controlled trial. | Du H, Newton PJ, Budhathoki C, Everett B, Salamonson Y, Macdonald PS, Davidson PM. | 2018 | Intervention |
| A nurse-led intervention to promote physical activity in sedentary older adults with cardiovascular risk factors: a randomized clinical trial (STEP-IT-UP study) | Chudowolska-Kielkowska M and Malek MA. | 2020 | Intervention |
| A randomized controlled trial of telephone-mentoring with home-based walking preceding rehabilitation in COPD. | Cameron-Tucker HL, Wood-Baker R, Joseph L, Walters JA, Schuz N, Walters EH. | 2016 | Intervention |
| Mediators of physical activity change in a behavioral modification program for type 2 diabetes patients | Van Dyck D, De Greef K, Deforche B, Ruige J, Tudor-Locke C.E, Kaufman J.-M, Owen N, De Bourdeaudhuij I. | 2011 | Comparator |
| Selected as the best paper in the 1990s: Reducing frailty and falls in older persons: an investigation of tai chi and computerized balance training. | Wolf SL, Barnhart HX, Kutner NG, McNeely E, Coogler C, Xu T, Atlanta FICSIT Group. | 2003 | Intervention |
| Effects of telerehabilitation on physical function and disability for stroke patients: a randomized, controlled trial. | Chumbler NR, Quigley P, Li X, Morey M, Rose D, Sanford J, Griffiths P, Hoenig H | 2012 | Intervention |
| App-based supplemental exercise during inpatient orthopaedic rehabilitation increases activity levels: A pilot randomised control trial | Bui T, King C, Llado A, Lee D, Leong G, Paraparum A, Li I, Scrivener K. | 2019 | Setting |
| An Innovative STRoke Interactive Virtual thErapy (STRIVE) Online Platform for Community-Dwelling Stroke Survivors: A Randomized Controlled Trial. | Johnson L, Bird M-L, Muthalib M, Teo W-P. | 2020 | Intervention |
| Tele-Health Followup Strategy for Tight Control of Disease Activity in Rheumatoid Arthritis: Results of a Randomized Controlled Trial. | de Thurah A, Stengaard-Pedersen K, Axelsen M, Fredberg U, Schougaard LMV, Hjollund NHI, Pfeiffer-Jensen M, Laurberg TB, Tarp U, Lomborg K, Maribo T. | 2018 | Intervention |
| Wristband Accelerometers to motiVate arm Exercises after Stroke (WAVES): a pilot randomized controlled trial. | Da-Silva RH, Moore SA, Rodgers H, Shaw L, Sutcliffe L, van Wijck F, Price CI. | 2019 | Setting |
| Face-to-face interaction compared with video watching on use of physical activity in peripheral arterial disease: A pilot trial | Collins T.C, Krueger P.N, Kroll T.L, Sharf B.F. | 2009 | Intervention |
| Tele-monitoring reduces exacerbation of COPD in the context of climate change-a randomized controlled trial | Jehn M, Donaldson G, Kiran B, Liebers U, Mueller K, Scherer D, Endlicher W, Witt C. | 2013 | Intervention |
| Short- and long-term effects of a physical activity counselling programme in COPD: A randomized controlled trial | Altenburg W.A, Ten Hacken N.H.T, Bossenbroek L, Kerstjens H.A.M, De Greef M.H.G, Wempe J.B. | 2015 | Intervention |
| The effectiveness of extended care based on Internet and home care platform for orthopaedics after hip replacement surgery in China. | Wang J, Tong Y, Jiang Y, Zhu H, Gao H, Wei R, Que X, Gao L. | 2018 | Mean age < 60 |
| The differentiated effectiveness of a printed versus a Web-based tailored physical activity intervention among adults aged over 50. | Peels DA, van Stralen MM, Bolman C, Golsteijn RHJ, de Vries H, Mudde AN, Lechner L. | 2014 | Comparator |
| Changes in Weight and Health-Related Behavior Using Smartphone Applications in Patients with Colorectal Polyps. | Lee K-W, Kim H-B, Lee S-H, Ha H-K. | 2019 | Mean age < 60 |
| Adding web-based behavioural support to exercise referral schemes for inactive adults with chronic health conditions: the e-coacher RCT | Taylor AH, Taylor TD, Ingram EM, Anokye N, Dean S, Jolly K, Mutrie N, Lambert J, Yardley L, Greaves C, King J, McAdam C, Steele M, Price L, Streeter A, Charles N, Terry R, Webb D, Campbell J, Hughes L, Ainsworth B, Jones B, Jane B, Erwin J, Little P, Woolf A, Cavanagh C. | 2020 | Mean age < 60 |
| A technology-assisted health coaching intervention vs. enhanced usual care for Primary Care-Based Obesity Treatment: A randomized controlled trial | Viglione C, Bouwman D, Rahman N, Fang Y, Beasley J.M, Sherman S, Pi-Sunyer X, Wylie-Rosett J, Tenner C, Jay M. | 2019 | Mean age < 60 |
| Effectiveness of tailored lifestyle interventions, using web-based and print-mail, for reducing blood pressure among rural women with prehypertension: main results of the Wellness for Women: DASHing towards Health clinical trial. | Hageman PA, Pullen CH, Hertzog M, Boeckner LS. | 2014 | Mean age < 60 |
| Effects of Computerized Cognitive Training and Tai Chi on Cognitive Performance in Older Adults with Traumatic Brain Injury | Hwang H.-F, Chen C.-Y, Wei L, Chen S.-J, Yu W.-Y, Lin M.-R. | 2019 | Duplicate |
| "WALK30X5": a feasibility study of a physiotherapy walking programme for people with mild to moderate musculoskeletal conditions. | Minns Lowe CJ, Kelly P, Milton K, Foster C, Barker K. | 2020 | Mean age < 60 |
| Impact of Online Weight Management with Peer Coaching on Physical Activity Levels of Adults with Serious Mental Illness. | Muralidharan A, Niv N, Brown CH, Olmos-Ochoa TT, Fang LJ, Cohen AN, Kreyenbuhl J, Oberman RS, Goldberg RW, Young AS. | 2018 | Study design |
| Changes in Moderate Intensity Physical Activity Are Associated with Better Cognition in the Multilevel Intervention for Physical Activity in Retirement Communities (MIPARC) Study | Zlatar Z.Z, Godbole S, Takemoto M, Crist K, Sweet C.M.C, Kerr J, Rosenberg D.E. | 2019 | Comparator |
| Health Promotion in Older Chinese: A 12-Month Cluster Randomized Controlled Trial of Pedometry and "Peer Support" | Thomas GN, Macfarlane DJ, Boliang G, Cheung BMY, McGhee SM, Chou K-L, Deeks JJ, Lam TH, Tomlinson B. | 2012 | Duplicate |
| Changes in postural balance in frail elderly women during a 4-week visual feedback training: a randomized controlled trial. | Sihvonen SE, Sipila S, Era PA. | 2004 | Intervention |
| It's LiFe! Mobile and Web-Based Monitoring and Feedback Tool Embedded in Primary Care Increases Physical Activity: A Cluster Randomized Controlled Trial. | van der Weegen S, Verwey R, Spreeuwenberg M, Tange H, van der Weijden T, de Witte L. | 2015 | Mean age < 60 |
| Community-based randomized controlled trial of diabetes prevention study for high-risk individuals of type 2 diabetes: lifestyle intervention using web-based system. | Cha S-A, Lim S-Y, Kim K-R, Lee E-Y, Kang B, Choi Y-H, Yoon K-H, Ahn Y-B, Lee J-H, Ko S-H. | 2017 | Study design |
| Health Promotion in Older Chinese: A 12-Month Cluster Randomized Controlled Trial of Pedometry and 'Peer Support'. | Thomas GN, Macfarlane DJ, Boliang G, Cheung BMY, McGhee SM, Chou K-L, Deeks JJ, Lam TH, Tomlinson B. | 2012 | Comparator |
| Effects of Computerized Cognitive Training and Tai Chi on Cognitive Performance in Older Adults with Traumatic Brain Injury. | Hwang H-F, Chen C-Y, Wei L, Chen S-J, Yu W-Y, Lin M-R. | 2020 | Intervention |
| A randomized control trial feasibility evaluation of an mHealth intervention for wheelchair skill training among middle-aged and older adults | Giesbrecht EM and Miller WC. | 2017 | Comparator |
| A randomized controlled trial of a wearable technology-based intervention for increasing moderate to vigorous physical activity and reducing sedentary behavior in breast cancer survivors: The ACTIVATE Trial. | Lynch BM, Nguyen NH, Moore MM, Reeves MM, Rosenberg DE, Boyle T, Vallance JK, Milton S, Friedenreich CM, English DR. | 2019 | Comparator |
| Psychosocial mediators of physical activity change in a web-based intervention for Latinas | Larsen B, Dunsiger S.I, Pekmezi D, Linke S, Hartman S.J, Marcus B.H. | 2021 | Mean age < 60 |
| Effect of Collaborative Telerehabilitation on Functional Impairment and Pain Among Patients with Advanced-Stage Cancer: A Randomized Clinical Trial. | Cheville AL, Moynihan T, Herrin J, Loprinzi C, Kroenke K. | 2019 | Comparator |
| Long-term efficacy of a printed or a Web-based tailored physical activity intervention among older adults. | Peels AD, Bolman C, Golsteijn RHJ, de Vries H, Mudde AN, van Stralen MM, Lechner L. | 2013 | Comparator |
| Effects of a randomized exercise trial on physical activity, psychological distress and quality of life in older adults. | Awick EA, Ehlers DK, Aguiñaga S, Daugherty AM, Kramer AF, McAuley E. | 2017 | Intervention |
| Effects of a Home-Based DVD-Delivered Physical Activity Program on Self-Esteem in Older Adults: Results from a Randomized Controlled Trial. | Awick EA, Ehlers D, Fanning J, Phillips SM, Wojcicki T, Mackenzie MJ, Motl R, McAuley E. | 2017 | Comparator |
| The Effects of a Telehealth Coping Skills Intervention on Outcomes in Chronic Obstructive Pulmonary Disease: Primary Results From the INSPIRE-II Study. | Blumenthal JA, Emery CF, Smith PJ, Keefe FJ, Welty-Wolf K, Mabe S, Martinu T, Johnson JJ, Babyak MA, O'Hayer VF, Diaz PT, Durheim M, Baucom D, Palmer SM. | 2014 | Intervention |
| Effectiveness and neural mechanisms of home-based telerehabilitation in patients with stroke based on fMRI and DTI | Chen J, Liu M, Sun D, Jin Y, Wang T, Ren C. | 2018 | Study design |
| Benefit of wearing an activity tracker in sarcoidosis | Drent M, Elfferich M, Breedveld E, De Vries J, Strookappe B. | 2020 | Mean age < 60 |
| Smartphone-Based Visual Feedback Trunk Control Training Using a Gyroscope and Mirroring Technology for Stroke Patients. | Shin DC and Song CH. | 2016 | Mean age < 60 |
| Complex Versus Simple Ankle Movement Training in Stroke Using Telerehabilitation: A Randomized Controlled Trial. | Deng H, Durfee WK, Nuckley DJ, Rheude BS, Severson AE, Skluzacek KM, Spindler KK, Davey CS, Carey JR.. | 2012 | Comparator |
| The Effects of an Online Theory-Based Bone Health Program for Older Adults. | Nahm E-S, Resnick B, Brown C, Zhu S, Magaziner J, Bellantoni M, Brennan PF, Charters K, Brown J, Rietschel M, An M, Park BK. | 2017 | Comparator |
| Acceptability of a mobile health exercise-based cardiac rehabilitation intervention: A randomized trial | Pfaeffli DL, Whittaker R, Dixon R, Stewart R, Jiang Y, Carter K, Maddison R. | 2015 | Outcomes |
| Internet-delivered therapist-guided physical activity for mild to moderate depression: A randomized controlled trial | Strom M, Uckelstam C-J, Andersson G, Hassmen P, Umefjord G, Carlbring P. | 2013 | Mean age < 60 |
| Interactive web-based pulmonary rehabilitation programme: a randomised controlled feasibility trial. | Chaplin E, Hewitt S, Apps L, Bankart J, Pulikottil-Jacob R, Boyce S; Morgan M, Williams J, Singh S. | 2017 | Intervention |
| Effects of Pedometer Use in Veterans with Chronic Heart Failure. | Chang VM, Dallas MI, Lampley TM, Milner KA. | 2015 | Intervention |
| Online versus face-to-face pulmonary rehabilitation for patients with chronic obstructive pulmonary disease: randomised controlled trial. | Bourne S, DeVos R, North M, Chauhan A, Green B, Brown T, Cornelius V, Wilkinson T. | 2017 | Intervention |
| Weight management telehealth intervention for overweight and obese rural cardiac rehabilitation participants: A randomised trial. | Barnason S, Zimmerman L, Schulz P, Pullen C, Schuelke S. | 2019 | Intervention |
| Impact of a home communication intervention for coronary artery bypass graft patients with ischemic heart failure on self-efficacy, coronary disease risk factor modification, and functioning. | Barnason S, Zimmerman L, Nieveen J, Schmaderer M, Carranza B, Reilly S. | 2003 | Intervention |
| Using interactive Internet technology to promote physical activity in Latinas: Rationale, design, and baseline findings of Pasos Hacia La Salud | Marcus B.H, Hartman S.J, Pekmezi D, Dunsiger S.I, Linke S.E, Marquez B, Gans K.M, Bock B.C, Larsen B.A, Rojas C. | 2015 | Mean age < 60 |
| A Walking Intervention to Increase Weekly Steps in Dialysis Patients: A Pilot Randomized Controlled Trial. | Sheshadri A, Kittiskulnam P, Lazar AA, Johansen KL. | 2020 | Comparator |
| Tailored Therapist-Guided Internet-Based Cognitive Behavioral Treatment for Psoriasis: A Randomized Controlled Trial. | van Beugen S, Ferwerda M, Spillekom-van Koulil S, Smit JV, Zeeuwen-Franssen MEJ, Kroft, EBM, de Jong EMGJ, Otero ME, Donders ART, van de Kerkhof PCM, van Middendorp HA, Evers AWM. | 2016 | Mean age < 60 |
| Increases in muscle strength and balance using a resistance training program administered via a telecommunications system in older adults. | Sparrow D, Gottlieb DJ, Demolles D, Fielding RA. | 2011 | Comparator |
| Supported community exercise in people with long-term neurological conditions: A phase II randomized controlled trial | Elsworth C, Winward C, Sackley C, Meek C, Freebody J, Esser P, Hooshang I, Soundy A, Barker K, Hilton-Jones D | 2011 | Mean age < 60 |
| Effects of tailoring health messages on physical activity. | Smeets T, Brug J, de Vries H. | 2008 | Mean age < 60 |
| Feasibility and preliminary effects of a peer-led motivationally embellished workplace walking intervention: A pilot cluster randomized trial (the START trial) | Thogersen-Ntoumani C, Quested E, Smith B.S, Nicholas J, McVeigh J, Fenton S.A.M, Stamatakis E, Parker S, Pereira G, Gucciardi D.F, Ntoumanis N. | 2020 | Mean age < 60 |
| Randomized controlled trial of a computer-tailored multiple health behaviour intervention in general practice: 12-month follow-up results. | Parekh S, King D, Boyle FM, Vandelanotte C. | 2014 | Mean age < 60 |
| Effectiveness of a Smartphone Application as a Support Tool for Patients Undergoing Breast Cancer Chemotherapy: A Randomized Controlled Trial | Handa S, Okuyama H, Yamamoto H, Nakamura S, Kato Y. | 2020 | Mean age < 60 |
| Comparing a Video and Text Version of a Web-Based Computer-Tailored Intervention for Obesity Prevention: A Randomized Controlled Trial. | Walthouwer MJL, Oenema A, Lechner L, de Vries H. | 2015 | Mean age < 60 |
| Utilization of short message service (SMS) in non-pharmacological management of hypertension. A pilot study in an URBAN public hospital of Multan, Pakistan. | Rehman A, Naeem FI, Abbas S, Ashfaq F, Hassali M. | 2019 | Comparator |
| Motivation and its relationship to adherence to self-monitoring and weight loss in a 16-week Internet behavioral weight loss intervention. | Webber KH, Tate DF, Ward DS, Bowling JM. | 2010 | Mean age < 60 |
| Effects of interactive visual feedback training on post-stroke pusher syndrome: a pilot randomized controlled study. | Yang Y-R, Chen Y-H, Chang H-C, Chan R-C, Wei S-H, Wang R-Y. | 2015 | Intervention |
| A pragmatic randomised controlled trial of the effectiveness and cost-effectiveness of 'PhysioDirect' telephone assessment and advice services for physiotherapy. | Salisbury C, Foster N, Hopper C, Bishop A, Hollinghurst S, Coast J, Kaur S, Pearson J, Franchini A, Hall J, Grove S, Calnan M, Busby J, Montgomery A. | 2013 | Intervention |
| Effectiveness of a smartphone application for weight loss compared with usual care in overweight primary care patients | Laing B.Y, Mangione C.M, Tseng C.-H, Leng M, Vaisberg E, Mahida M, Bholat M, Glazier E, Morisky D.E, Bell D.S. | 2014 | Mean age < 60 |
| Randomized trial of a phone- and web-based weight loss program for women at elevated breast cancer risk: the HELP study. | Cadmus-Bertram L, Nelson S, Hartman S, Patterson R, Parker B, Pierce J. | 2016 | Comparator |
| Building a physical activity intervention into clinical care for breast and colorectal cancer survivors in Wisconsin: a randomized controlled pilot trial. | Cadmus-Bertram L, Tevaarwerk AJ, Sesto ME, Gangnon R, Van Remortel B, Date P. | 2019 | Mean age < 60 |
| Randomised controlled feasibility study of the MyHealthAvatar-Diabetes smartphone app for reducing prolonged sitting time in type 2 diabetes mellitus | Bailey D.P, Mugridge L.H, Dong F, Zhang X, Chater A.M. | 2020 | Mean age < 60 |
| Can multiple lifestyle behaviours be improved in people with familial hypercholesterolemia? Results of a parallel randomised controlled trial. | Broekhuizen K, van Poppel MNM, Koppes LL, Kindt I, Brug J, van Mechelen W. | 2012 | Mean age < 60 |
| Improved early outcome after TKA through an app-based active muscle training programme-a randomized-controlled trial. | Hardt S, Schulz MRG, Pfitzner T, Wassilew G, Horstmann H, Liodakis E, Weber-Spickschen TS. | 2018 | Setting |
| Evaluation of a very brief pedometer-based physical activity intervention delivered in NHS Health Checks in England: The VBI randomised controlled trial. | Hardeman W, Mitchell J, Pears S, Van Emmenis M, Theil F, Gc VS, Vasconcelos JC, Westgate K, Brage S, Suhrcke M, Griffin SJ, Kinmonth AL, Wilson ECF, Prevost AT, Sutton S. | 2020 | Mean age < 60 |
| Exercise and internet-based cognitive-behavioural therapy for depression: multicenter randomised controlled trial with 12-month follow-up. | Hallgren M, Helgadottir B, Herring MP, Zeebari Z, Lindefors N, Kaldo V, Öjehagen A, Forsell Y. | 2016 | Mean age < 60 |
| Effects of a Web-Based Personalized Intervention on Physical Activity in European Adults: A Randomized Controlled Trial. | Marsaux CF, Celis-Morales C, Fallaize R, Macready AL, Kolossa S, Woolhead C, O’Donovan CB, Forster H, Navas-Carretero S, San-Cristobal R, Lambrinou C-P, Moschonis G, Surwillo A, Godlewska M, Goris A, Hoonhout J, Drevon CA, Manios Y, Traczyk I, Walsh MC, Gibney ER, Brennan L, Martinez JA, Lovegrove JA, Gibney MJ, Daniel H, Mathers JC, Saris WH. | 2015 | Mean age < 60 |
| Optimising Web-Based Computer-Tailored Physical Activity Interventions for Prostate Cancer Survivors: A Randomised Controlled Trial Examining the Impact of Website Architecture on User Engagement. | Finlay A, Evans H, Vincent A, Wittert G, Vandelanotte C, Short CE. | 2020 | Comparator |
| What is the comparative effectiveness of current standard treatment, against an individually tailored behavioural programme delivered either on the Internet or face-to-face for people with acute whiplash associated disorder? A randomized controlled trial. | Bring A, Åsenlöf P, Söderlund A. | 2016 | Mean age < 60 |
| Active8! Technology-Based Intervention to Promote Physical Activity in Hospital Employees. | Blake H, Suggs LS, Coman E, Aguirre L, Batt ME. | 2017 | Mean age < 60 |
| The impact of a home-based computerized cognitive training intervention on fall risk measure performance in community dwelling older adults, a pilot study. | Blackwood, J, Shubert, T, Fogarty, K, Chase, C. | 2016 | Intervention |
| Home-based telerehabilitation in older patients with chronic obstructive pulmonary disease and heart failure: a randomised controlled trial. | Bernocchi P, Vitacca M, La Rovere MT, Volterrani M, Galli T, Baratti D, Paneroni M, Campolongo G, Sposato B, Scalvini S. | 2018 | Comparator |
| Regular use of pedometer does not enhance beneficial outcomes in a physical activity intervention study in type 2 diabetes mellitus | Bjorgaas M.R, Vik J.T, Stolen T, Lydersen S, Grill V. | 2008 | Outcomes |
| Home-based telerehabilitation in older patients with chronic obstructive pulmonary disease and heart failure: a randomised controlled trial. | Bernocchi P, Vitacca M, La Rovere MT, Volterrani M, Galli T, Baratti D, Paneroni M, Campolongo G, Sposato B, Scalvini S. | 2018 | Duplicate |
| Impact of ENHANCED (diEtitiaNs Helping pAtieNts CarE for Diabetes) Telemedicine Randomized Controlled Trial on Diabetes Optimal Care Outcomes in Patients with Type 2 Diabetes. | Benson GA, Sidebottom A, Hayes J, Miedema MD, Boucher J, Vacquier M, Sillah A, Gamam S, VanWormer JJ. | 2019 | Intervention |
| Telephone Coaching to Enhance a Home-Based Physical Activity Program for Knee Osteoarthritis: A Randomized Clinical Trial. | Bennell KL, Campbell PK, Egerton T, Metcalf B, Kasza J, Forbes A, Bills C, Gale J, Harris A, Kolt GS, Bunker SJ, Hunter DJ, Brand CA, Hinman RS. | 2017 | Intervention |
| The Rehabilitation Enhancing Aging Through Connected Health Prehabilitation Trial. | Bean JF, Brown L, DeAngelis TR, Ellis T, Kumar VSS, Latham NK, Lawler D, Ni M, Perloff J. | 2019 | Study design |
| Experience of Using an App in HIV Patients Older Than 60 Years: Pilot Program. | Olalla J, Garcia de Lomas JM, Marquez E, Gonzalez FJ, Del Arco A, De La Torre J, Prada JL, Cantudo F, Martin MD, Nieto M, Perez Stachowski J Garcia-Alegria. | 2019 | Study design |
| FOOTFIT Physical Activity mHealth Intervention for Minimally Ambulatory Individuals with Venous Leg Ulcers: A Randomized Controlled Trial. | Kelechi TJ, Madisetti M, Prentice M, Mueller M. | 2020 | Comparator |
| Randomized controlled pilot of an intervention to reduce and break-up overweight/obese adults' overall sitting-time. | Júdice PB, Hamilton MT, Sardinha LB, Silva AM. | 2015 | Mean age < 60 |
| E-mail-based exercises in happiness, physical activity and readings: A randomized trial on 3274 Finns | Joutsenniemi K, Kaattari C, Harkanen T, Pankakoski M, Langinvainio H, Lonnqvist J, Mattila A.S, Mustonen P. | 2014 | Mean age < 60 |
| Mobile Health Management Platform-Based Pulmonary Rehabilitation for Patients with Non-Small Cell Lung Cancer: Prospective Clinical Trial. | Ji W, Kwon H, Lee S, Kim S, Sook Hong J, Rang Park Y, Ryul Kim H, Lee JC, Jung EJ, Kim D, Choi C-M. | 2019 | Mean age < 60 |
| Effects of Mobile Text Messaging on Glycemic Control in Patients with Coronary Heart Disease and Diabetes Mellitus: A Randomized Clinical Trial. | Huo X, Krumholz HM, Bai X, Spatz ES, Ding Q, Horak P, Zhao W, Gong Q, Zhang H, Yan X, Sun Y, Liu J, Wu X, Guan W, Wang X, Li J, Li X, Spertus JA, Masoudi FA, Zheng X. | 2019 | Mean age < 60 |
| Comparison of two short-term balance training programs for community-dwelling older adults | Hinman MR | 2002 | Intervention |
| Effects of traditional physical training and visual computer feedback training in frail elderly patients. A randomized intervention study | Hagedorn DK and Holm E. | 2010 | Intervention |
| The impact of an online social network with wireless monitoring devices on physical activity and weight loss. | Greene J, Sacks R, Piniewski B, Kil D, Hahn JS. | 2013 | Mean age < 60 |
| Waste the waist: a pilot randomised controlled trial of a primary care-based intervention to support lifestyle change in people with high cardiovascular risk. | Greaves C, Gillison F, Stathi A, Bennett P, Reddy P, Dunbar J, Perry R, Messom D, Chandler R, Francis M, Davis M, Green C, Evans P, Taylor G. | 2015 | Intervention |
| Outcomes of minimal and moderate support versions of an internet-based diabetes self-management support program. | Glasgow RE, Kurz D, King D, Dickman JM, Faber AJ, Halterman E, Wooley T, Toobert DJ, Strycker LA, Estabrooks PA, Osuna D, Ritzwoller D. | 2010 | Mean age < 60 |
| Feasibility of a home-based exercise intervention with remote guidance for patients with stable grade II and III gliomas: a pilot randomized controlled trial. | Gehring K, Kloek CJ, Aaronson NK, Janssen KW, Jones LW, Sitskoorn MM, Stuiver MM. | 2018 | Mean age < 60 |
| Randomised controlled trial of a pedometer-based telephone intervention to increase physical activity among cardiac patients not attending cardiac rehabilitation | Furber S, Butler L, Phongsavan P, Mark A, Bauman A. | 2010 | Comparator |
| The "Moving Heart Program": an intervention to improve physical activity among patients with coronary heart disease1. | Rodrigues RCM, São João TM, Gallani MCBJ, Cornélio ME, Alexandre NMC. | 2013 | Mean age < 60 |
| An education program for risk factor management after an acute coronary syndrome: a randomized clinical trial. | Cohen A, Assyag P, Boyer-Chatenet L, Cohen-Solal A, Perdrix C, Dalichampt M, Michel P-L, Montalescot G, Ravaud P, Steg PG, Boutron I, Réseau Insuffisance Cardiaque (RESICARD) PREVENTION Investigators | 2014 | Mean age < 60 |
| Web-based self-monitoring for weight loss among overweight/obese women at increased risk for breast cancer: the HELP pilot study. | Cadmus-Bertram l, Wang JB, Patterson RE, Newman VA, Parker BA, Pierce JP. | 2013 | Comparator |
| Investigating the behavioural effects of a mobile-phone based home telehealth intervention in people with insulin-requiring diabetes: Results of a randomized controlled trial with patient interviews. | Baron JS, Hirani SP, Newman SP. | 2017 | Mean age < 60 |
| Chronic obstructive pulmonary disease self-management activation research trial (COPD-SMART): design and methods. | Ashmore J, Russo R, Peoples J, Sloan J, Jackson BE, Bae S, Singh KP, Blair SN, Coultas D. | 2013 | Study design |
| Outpatient physiotherapy versus home-based rehabilitation for patients at risk of poor outcomes after knee arthroplasty: CORKA RCT. | Barker KL, Room J, Knight R, Dutton SJ, Toye F, Leal J, Kent S, Kenealy N, Schussel MM, Collins G, Beard DJ, Price A, Underwood M, Drummond A, Cook E, Lamb SE. | 2020 | Intervention |
| Effects and barriers to deployment of telehealth wellness programs for chronic patients across 3 European countries. | Barberan-Garcia A, Vogiatzis I, Solberg HS, Vilaro J, Rodriguez DA, Garasen HM, Troosters T, Garcia-Aymerich J, Roca J, NEXES Consortium | 2014 | Study design |
| Efficacy of a pedometer-based physical activity program on parameters of diabetes control in type 2 diabetes mellitus | Araiza P, Hewes H, Gashetewa C, Vella C.A, Burge M.R. | 2006 | Mean age < 60 |
| An Internet- and mobile-based tailored intervention to enhance maintenance of physical activity after cardiac rehabilitation: short-term results of a randomized controlled trial. | Antypas K and Wangberg SC | 2014 | Mean age < 60 |
| Short-term and long-term cost-effectiveness of a pedometer-based exercise intervention in primary care: a within-trial analysis and beyond-trial modelling. | Anokye N, Fox-Rushby J, Sanghera S, Cook DG, Limb E, Furness C, Kerry SM, Victor CR, Iliffe S, Ussher M, Whincup PH, Ekelund U, deWilde S, Harris T. | 2018 | Study design |
| Integrating Mobile-health, health coaching, and physical activity to reduce the burden of chronic low back pain trial (IMPACT): a pilot randomised controlled trial. | Amorim AB, Pappas E, Simic M, Ferreira ML, Jennings M, Tiedemann A, Carvalho-e-Silva AP, Caputo E, Kongsted A, Ferreira PH. | 2019 | Mean age < 60 |
| A randomized controlled trial of weight reduction and exercise for diabetes management in older African American subjects. | Agurs-Collins TD, Kumanyika SK, Ten Have TR, Adams-Campbell LL, Agurs-Collins TD, Kumanyika SK, Have TR, Adams-Campbell LL. | 1997 | Intervention |
| Efficacy of the Type 2 Diabetes Prevention Using LifeStyle Education Program RCT | Aguiar E.J, Morgan P.J, Collins C.E, Plotnikoff R.C, Young M.D, Callister R. | 2016 | Mean age < 60 |
| A daily physical activity and diet intervention for individuals with type 2 diabetes mellitus: a randomized controlled trial. | Van Rooijen AJ, Christa MV, Piet JB. | 2010 | Mean age < 60 |
| A multilevel approach for promoting physical activity in rural communities: a cluster randomized controlled trial. | Beck AM, Eyler AA, Hipp JA, King AC, Tabak RG, Yan Y, Reis RS, Duncan DD, Gilbert AS, Serrano NH, Brownson RC. | 2019 | Study design |
| Effectiveness of A Multifactorial Intervention in Increasing Adherence to the Mediterranean Diet among Patients with Diabetes Mellitus Type 2: A Controlled and Randomized Study (EMID Study). | Alonso-Dominguez R, Garcia-Ortiz L, Patino-Alonso MC, Sanchez-Aguadero N, Gomez-Marcos MA, Recio-Rodriguez JI. | 2019 | Comparator |
| The effect of innovative smartphone application on adherence to a home-based exercise program for female older adults with knee osteoarthritis in Saudi Arabia: a randomized controlled trial | Alasfour M and Almarwani M. | 2020 | Mean age < 60 |
| Lifestyle interventions in Muslim patients with metabolic syndrome-a feasibility study | Aktas M.F, Mahler A, Hamm M, Perger G, Simon F, Westenhofer J, Luft F.C, Boschmann M. | 2019 | Mean age < 60 |
| A one-way text messaging intervention for obesity. | Ahn A and Choi J. | 2016 | Mean age < 60 |
| International Journal of Behavioral Nutrition and Physical Activity The effectiveness of a web 2.0 physical activity intervention in older adults - a randomised controlled trial. | Alley SJ, Kolt GS, Duncan MJ, Caperchione CM, Savage TN, Maeder AJ, Rosenkranz RR, Tague R, Van Itallie AK, Mummery WK, Vandelanotte C. | 2018 | Duplicate |
| The effectiveness of a web 2.0 physical activity intervention in older adults - a randomised controlled trial. | Alley SJ, Kolt GS, Duncan MJ, Caperchinoe CM, Savage TN, Maeder AJ, Rosenkranz RR, Tague R, Van Itallie AK, Mummery WK, Vandelanotte C. | 2018 | Mean age < 60 |
| Diabetes and TelecommunicationS (DATES) study to support self-management for people with type 2 diabetes: a randomized controlled trial. | Al-Ozairi E, Ridge K, Taghadom E, de Zoysa N, Tucker C, Stewart K, Stahl D, Ismail K. | 2018 | Study design |

# **3. Detailed intervention descriptions**

## Table 6. Intervention and control group descriptions

| **Study** | **Intervention** | **Control/Usual Care** |
| --- | --- | --- |
| Alonso-Dominguez et al [41], 2019  [smart-phone application] | Participants underwent a multifactorial intervention consisting of aerobic walks, diet workshop, and use of a smartphone application. The interventions are described below and were guided by nurses from the health center.  *Hearty-healthy walks:* Once per week for five weeks, participants walked 4 km on level ground at 50-70% of maximum heart rate. These walks were preceded by warm-up exercises for 10-mins and followed by 10-mins of stretching and relaxation. While all participants completed these aerobic walks, they were subdivided into moderate intensity (5 metabolic equivalents (METs), walking at 6 km/hr) and low intensity (2.5 METs, walking at 3-4 km/hr) groups.  *Smartphone application:* Participants were provided a smartphone and a one-hour instructional workshop on the EVIDENT II application, which is designed to increase physical activity and adherence to the Mediterranean diet. The application, which is individually configured with participants’ age, sex, weight, and height, tailors advice to encourage increased levels of physical activity (if needed and based on daily PA assessments from the application) and healthier eating choices. Participants could manually enter PA data when the device could not be used, for example, with swimming.  Participants in the intervention group also received usual care as described. | Usual care was provided by three nurses at the health center who had been previously trained to deliver the counselling. Sessions consisted of a standardized 10-minutes of counselling covering topics on physical activity and healthy eating. Specifically, physical activity was covered for 5-minutes and included guidance on how to adhere to current PA recommendations (i.e., walk 10,000 steps/day and avoid sedentary behaviors). Counselling on diet encompassed the remaining 5-minutes and covered topics such as how to adhere to the Mediterranean diet. Written materials covering these topics were provided to participants. |
| Armit et al [48], 2005  [Pedometer] | Participants in this 12-week intervention participated in several components, including attending a single 15-20-min counselling session tailored to the individual to encourage PA and increase daily step counts. These sessions occurred within 5-days of recruitment. In addition, they received an information booklet, and a diary for goal setting and self-monitoring. Over the course of the 12-week intervention, participants received three 12-15-min telephone calls to review and re-evaluate the goals, reinforce positive behaviors, and discuss any challenges with adherence. Finally, participants in this group received a pedometer to track daily steps. | Usual care participants received brief verbal advice (3-5 mins) from a general practitioner, a written prescription for PA, and an information booklet.  *Usual care arm 1:* Participants received brief verbal advice (3-5 mins) from a general practitioner, a written prescription for PA, and an information booklet.  *Usual care arm 2:* Participants attended a single counselling session delivered by an exercise scientist. The session was 15-20 mins long and was tailored to the participant to encourage PA. These sessions occurred within 5-days of recruitment. They also received an information booklet, in addition to a diary for goal setting and self-monitoring. Finally, over the course of the 12-week intervention, participants received three 12-15 min telephone calls to re-evaluate goals, reinforce positive behaviors, and discuss any issues with adherence. |
| Audsley et al [49], 2020  [Pedometer] | Participants engaged in six motivational interviewing and behavior change technique group sessions that lasted 60-90-mins delivered over the course of 6-months. The content of sessions included knowledge of PA, information about local PA services, planning weekly activities, overcoming barriers, problem-solving, identifying relapse prevention strategies, building new habits, stress management, and self-regulation skills.  In addition to these sessions, participants received a pedometer and an instructional manual containing illustrated exercises, worksheets, and PA diaries. If a participant was unable to attend in person, the information was delivered by telephone. Participants in this group also had access to usual care.  Personnel delivering the Keeping Adults Physically Active (KAPA) program were trained in motivational interviewing and standard operating procedures for this program. | Usual care consisted of a 24-week Falls Management Exercise (FaME) class offered weekly. FaME contains age-specific strength, balance, cardiovascular, and flexibility exercises aimed at reducing the risk of falls and improving physical function in older adults. In addition to these, group classes included training participants how to get up from, and down to, the floor, and coping strategies to reduce the risk of complications resulting from inactivity and associated complications. Classes were delivered in a group setting by a postural stability instructor. In addition to these classes, participants were given a 30-min home exercise program of leg muscle strengthening and balance retraining to be completed twice per week. Participants received an instruction booklet for support. Finally, participants were advised to walk two times per week at a moderate pace for up to 30-mins. |
| Barnason et al [37], 2009  [Telerehabil-itation] | Participants received a 6-week (42 daily sessions) symptom management (SM) telehealth intervention designed to improve participants’ self-efficacy and self-management of early recovery symptoms. The intervention was delivered by the Health Buddy ® device and guided participants through self-management strategies to address common symptoms experienced during recovery from coronary artery bypass surgery. Strategies addressed topics such as rest, pain management, and progression of physical activity after surgery. The goal of the SM intervention was to improve patient outcomes and reduce healthcare utilization. The intervention group also received usual care. | Usual care was not defined. |
| Christiansen et al [43], 2020  [Wearable activity tracker] | Participants received usual care, which consisted of a physical therapy program (according to the Delaware Physical Therapy Clinic Rehab Practice Guidelines for Unilateral TKR) focused on range of motion, neuromuscular electrical stimulation, strength, and balance and agility. They also received a home exercise program focused on range of motion, strength, balance and agility, and a progressive walking program. Participants received a log to record their adherence that was updated weekly by the physiotherapist.  In addition, participants received a weekly steps/day goal set with the physiotherapist (to a minimum of 6,000 steps/day by end intervention), monthly follow-up phone calls for 6-months to promote PA, and a Fitbit Zip to track their activity. Participants were asked to wear the Fitbit Zip during waking hours and to monitor and record their steps/day count on the log in their home exercise program. | Control group participants received standard outpatient physiotherapy according to the Delaware Physical Therapy Clinic Rehab Practice Guidelines for Unilateral TKR. Rehabilitation was progressive over 10-weeks (up to 28 visits) and focused on range of motion, neuromuscular electrical stimulation, strength, and balance and agility.  In addition to this physical therapy program, participants received a printed home exercise program focused on range of motion/stretching, strength, balance and agility, and a walking program along with a log to record their adherence. The home program was updated weekly by the physiotherapist. Finally, participants received a monthly phone call for 6-months after discharge to review their overall health and remind them of the outcomes appointments at 6- and 12-months. These points of contact also matched the same number of contacts in the intervention group. |
| de Blok et al [32], 2006  [Pedometer] | Participants in both groups received regular pulmonary rehabilitation consisting of exercise training, dietary interventions, and education according to guidelines from the American College of Chest Physicians and the American Association of Cardiovascular and Pulmonary Rehabilitation for 9-weeks. In addition to this, participants in the intervention group received four 30-min lifestyle PA counselling sessions and received a pedometer to track and encourage PA. PA counselling consisted of motivation to increase PA in daily life activities (e.g., walking, cycling, stair-climbing, and gardening). Counselling was delivered by trained physiotherapists and followed the principles of motivational interviewing. Pedometers were worn for 10-weeks (1-week prior to rehab and 9-weeks during).  *Session 1* (2 weeks prior to rehabilitation): focused on motivation for increasing PA. Participants received their pedometers during this session.  *Session 2* (1^st^ week of rehab): consisted of goal-setting.  *Session 3* (5^th^ week of rehab): focused on shifting boundaries. In addition, participants were asked to set a goal to achieve their maximal PA limit once (measured in steps).  *Session 4* (7^th^ week of rehab): focused on consolidating PA behavior and goal-setting for their personal activity norm (expected to be between their mean steps/day and maximal steps). | Usual care was a 9-week pulmonary rehabilitation program consisting of exercise training, dietary interventions, and education according to guidelines from the American College of Chest Physicians and the American Association of Cardiovascular and Pulmonary Rehabilitation. |
| Frederix et al [38], 2015  [Multi-component] | Participants in the telerehabilitation program received an Internet-based program for 24-weeks in addition to the usual care center-based cardiac rehabilitation program. Telerehabilitation began on week 6 of the 12-week cardiac rehab, which facilitated an introduction and familiarization with the accelerometer prior to beginning the intervention. This program used PA telemonitoring with an accelerometer along with dietary, smoking cessation, and PA telecoaching strategies.  Participants received individualized exercise training programs based on their peak aerobic capacity (VO2 peak) and BMI. They were instructed to wear the accelerometer continuously and to regularly transmit their PA data. PA data transmission triggered a semiautomatic telecoaching system that provided once weekly feedback via email or text messaging. The feedback focused on encouraging increases in PA to achieve their predefined training goals, tailored dietary recommendations (including a module for diabetes, arterial hypertension, obesity, and health), and smoking cessation recommendations. The feedback modified over time based on the participants lifestyle behaviors and changes.  Center-based cardiac rehab consisted of 45 multidisciplinary rehabilitation sessions (at least 2 exercise training sessions/week) over 12-weeks. Participants were instructed to exercise for 45-60-mins per session to a target heart rate and/or workload that corresponded to their first ventilatory threshold and respiratory compensation point. Walking/running and/or cycling and arm cranking were used for endurance exercises. Participants also received counselling from a dietician to follow health diet guidelines, and a psychologist session to improve self-efficacy towards healthier lifestyle behaviors. | Center-based cardiac rehab consisted of 45 multidisciplinary rehabilitation sessions (at least 2 exercise training sessions/week) over 12-weeks. Participants were instructed to exercise for 45-60-mins per session to a target heart rate and/or workload that corresponded to their first ventilatory threshold and respiratory compensation point. Walking/running and/or cycling and arm cranking were used for endurance exercises. Participants also received counselling from a dietician to follow health diet guidelines, and a psychologist session to improve self-efficacy towards healthier lifestyle behaviors. |
| Hansen et al [33], 2020  **Smart-technology alone*  [Telerehabil-itation] | Participants in the intervention group received a supervised pulmonary telerehabilitation program three times per week for 10 weeks. They received the videoconference software system and single touch screen along with exercise equipment consisting of a step-box and pairs of dumbbells prior to the intervention. The sessions, which occurred three times/week and were 60-minutes long (35-mins exercise for a weekly total of 105-mins + 20-mins education for a weekly total of 60-mins), were supervised by a physiotherapist and respiratory nurses via webcam with groups of 4 to 8 participants.  *Exercises:* focused on larger muscle groups with half allocated to upper extremities and half to lower. Sessions included a 5-min warm-up followed by 6 exercises using body weight and/or dumbbells and step-box for load. Exercises were progressed by time and intensity as able.  Session supervisors completed exercise logs for each participant.  *Education:* the major topics covered included COPD and its treatment, signs of exacerbation and plans of action, medication and use of devices, PA and exercise, the importance of nutrition in COPD, smoking cessation, and anxiety management with relaxation techniques. | Participants in the control group received conventional pulmonary rehabilitation over 10-weeks. They attended twice weekly 60-min exercise sessions (total 120-mins weekly) in groups of 6 to 12 patients where they completed individually tailored PA supervised by two physiotherapists. They also received individualized education lasting 60-90-mins once per week following the exercise session. These were led by a trained respiratory nurse. In addition, a chest physician, physiotherapist, and dietician each led one of the 10 sessions.  *Exercises:* began with a 5-10-min warm-up followed by 20-30-mins of endurance training (e.g., walking, cycling, treadmill, circuit training), 20-30-mins resistance training (50% dedicated to upper and lower extremities each), and a 5-10-min cool-down.  *Education:* main topics covered included COPD and its treatment, smoking cessation, daily PA, nutrition, medication and use of devices, signs of exacerbation and action plan, and use of nebulizer and oxygen apparatuses. |
| Kawagoshi et al [34], 2015  [Pedometer] | Participants in the intervention group received a multidisciplinary home-based pulmonary rehabilitation program in addition to a pedometer to provide feedback on PA. Participants were supervised by a respiratory therapist every 2-weeks in-hospital; at that time, exercise intensity was re-evaluated and progressed as appropriate.  *Pulmonary rehabilitation:* consisted of breathing retraining (i.e., pursed-lip, diaphragmatic, and slow deep breathing), upper and lower limb exercises including COPD sitting calisthenics, respiratory muscle stretching exercises, walking for a minimum 15-mins (on the level), and inspiratory muscle training (set at 30-40% of the maximal inspiratory muscle force).  *Education:* participants received monthly education lasting 45-mins that covered topics about equipment use, nutrition, stress management, relaxation techniques, home exercises, and the benefits of pulmonary rehabilitation.  *Pedometer use:* participants were instructed to wear the pedometer daily for 1-year and received monthly feedback about their use by rehab staff. Participants were also encouraged to achieve 8,000 steps/day and received verbal reinforcement to increase their PA by rehab staff. | Participants in the control group received a multidisciplinary home-based pulmonary rehabilitation program. Participants were supervised by a respiratory therapist every 2-weeks in-hospital; at that time, exercise intensity was re-evaluated and progressed as appropriate.  *Pulmonary rehabilitation:* consisted of breathing retraining (i.e., pursed-lip, diaphragmatic, and slow deep breathing), upper and lower limb exercises including COPD sitting calisthenics, respiratory muscle stretching exercises, walking for a minimum 15-mins (on the level), and inspiratory muscle training.  *Education:* participants received monthly education lasting 45-mins that covered topics about equipment use, nutrition, stress management, relaxation techniques, home exercises, and the benefits of pulmonary rehabilitation. |
| King et al [47], 2020  **Smart-technology alone* [Telerehabil-itation] | Participants in the intervention group received an introductory session followed by up to 28 brief (10-15-min) 1:1 advisor sessions via a virtual advisor (embodied conversational agent) over the course of 1-year. In addition, participants were provided a pedometer and encouraged to use it to track steps. They also received a calendar to note appointments and to track steps and walking minutes.  *Advisor sessions:* the introductory session served to establish the participant’s PA history, goals, anticipated barriers and facilitators, and to co-create a weekly PA plan. Information provided during this session covered national PA guidelines, safety tips, community center resources, and training on the use of the participant calendar and use of the pedometer (including instruction to use it regularly). Subsequent sessions consisted of introductory dialogue, review of health status, review of steps/day and minutes walked since last session (using pedometer data), acknowledgement of successes, problem-solving, goal-setting, and summary and wrap-up. In general, participants received weekly sessions for the first 2-months, then twice/month for the remaining 10-months. | Participants in the control group similarly received 1:1 PA counselling (an introductory session and up to 28 brief follow-up sessions over 1-year), however, these sessions were delivered by a human advisor. Sessions were similar in content, duration, and distribution over the study period. |
| Kwan et al [46], 2020  [Smart-phone application] | Participants in the intervention group received both the conventional behaviour change intervention and the mHealth intervention for 12 weeks.    *Conventional behaviour change:* interventions were administered by the same person with a bachelor’s degree in psychology who received additional training in behaviour change theory and brisk walking. Health education began in the first week, and exercise training in brisk walking took place in weeks one and two. Training sessions took place in an elderly center and in a real environment such as a park. At baseline, interventions were tailored to each participant regarding their physical fitness. Exercise goals were set according to four principles: 1) practice availability, 2) baseline fitness, 3) previous performance, and 4) personal wish. Week three participants were trained on how to use a smartphone.  *Face-to-face meetings:* using WhatsApp and Samsung Health took place in weeks four, eight and 12.  *Follow up:* Follow up messages were completed using WhatsApp and Samsung Health, starting in the third week. Participants received messages at least once a week; topics of messages included, praise, e-reminders, personalized goals, or coaching. Messages were sent for the following reasons: 1) routine weekly message, 2) participant did not complete brisk walking for more than two days, and 3) weekly goal was achieved early. | The usual care group only received the conventional behaviour change intervention for 12 weeks.  *Conventional behaviour change:* interventions were administered by the same person with a bachelor’s degree in psychology who received additional training in behaviour change theory and brisk walking. Health education began in the first week, and exercise training in brisk walking took place in weeks one and two. Training session took place in an elderly center and in a real environment such as a park. At baseline, interventions were tailored to each participant regarding their physical fitness level. Exercise goals were set according to four principles: 1) practice availability, 2) baseline fitness, 3) previous performance, and 4) personal wish.  *Face-to-face meetings:* took place in weeks four, eight and 12. The intervention was tailored to each participant regarding their physical fitness level at baseline.  *Follow up:* the usual care group received two follow up phone calls during weeks six and ten. |
| Maddison et al [39], 2015  [Multi-component] | Participants were free to use any cardiac rehabilitation service or support over the course of the 24-week study. Cardiac rehabilitation in this area typically includes community-based education sessions on, cardiovascular disease risk factors, psychological support, physical activity encouragement, and an offer to join a local cardiac club that offers supervised exercise.  *Heart Programme:* the primary goal of this program was to have get individuals to perform moderate-to-vigorous aerobic activity for 30-minutes most days (at least 5 days). The program provided participants with regular exercise prescription, behaviour change strategies, and technical support.  *Text messages:* participants in the intervention group also received personalized, automated text messages via their mobile phones. They received three to five messages a week (for a total of 118 messages over the 24-week intervention).  *Website:* participants were encouraged to log in on a website to access a role model video vignettes (new videos were added weekly), ability to track their progress, and find information on forms of physical activity and exercise, energy expenditure, healthy eating, and links to external resources. | Participants were free to use any cardiac rehabilitation service or support over the course of the 24-week study. Cardiac rehabilitation in this area typically includes community-based education sessions on, cardiovascular disease risk factors, psychological support, physical activity encouragement, and an offer to join a local cardiac club that offers supervised exercise. |
| Mendoza et al [35], 2020  [Pedometer] | All participants attended counselling visit at the Hospital Clínico Universidad de Chile, delivered by their physician and physiotherapist. Thirty-minute visits occurred monthly for three months.  *Pedometer:* Participants received the PD724 Triaxial pedometer (Tanita, Tokyo, Japan) and were instructed on how to use it and to bring it to every visit.  *Activity diary*: Participants were instructed to record their daily steps counts in their diary along with any information related to their condition and bring this to each of their visits.  *Counselling:* participants in the pedometer group were encouraged to increase their step count based on the average number of steps taken a day in the previous week (taken from the pedometer at visit). Goals were given based on a predetermined protocol:  step count at monthly visit:  <6000 steps/day - increase by 3000 steps/day  >6000 and <9000steps/day - reach 9000 steps/day  >9000 steps/day - maintain or increase steps | All participants attended counselling visit at the Hospital Clínico Universidad de Chile, delivered by their physician and physiotherapist. Thirty-minute visits occurred monthly for three months.  *Activity diary:* Participants were instructed to record information related to their condition each day and bring this to each of their visits.  *Counselling:* participants in the control group were encouraged to increase their physical activity levels at each visit and advised to walk for 30-minutes each day. |
| Mouton and Cloes [50], 2015  **Smart-technology alone*  [Website] | The Move More intervention consisted of three intervention groups and a control. The primary focus of this intervention was to promote PA over a three-month period. Intervention groups included:   1. Web-based (website only) 2. Center-based (physical activity program center only) 3. Mixed (completed both the website and physical activity program)   *Website:* the website construction was informed by the transtheoretical model of health behaviour change and an ecological model to focus on both individual and environmental influences.  *Website sections:* 1) why should I move?, 2)What is physical activity, 3) what are the recommendations, 4) success stories, 5) useful links, 6) tips to start, 7) fixing goals, 8) overcome barriers, choose an activity, 10), exercise examples, 11) my physical activity journal, 12) tools to measure physical activity, 13) local physical activity opportunities, 14) local physical activity trails, 15) online forum, and 16) news.  *Tailored feedback:* participants completed a questionnaire at the beginning of each month (this was mandatory to maintain access to the website). Feedback was automatically generated and included: tips based on their stage of change, awareness of physical activity, local opportunities, were provided and suggestions for relevant website sections given.  *Physical activity program center:* this three-month physical activity program consisted of 12 group sessions (once a week). Program was created and delivered by a trained physical educators experienced with older adults.  *Exercise sessions:* sessions included: light cardiorespiratory, muscular and articular warm up, a combination of endurance, strength, flexibility and balance training, and light cool-down based on relaxation. Exercises given were safe for participant to complete at home with minimal equipment.  *Advice:* participants were also given motivational advice (e.g., create calendar or fix personal goals) and environmental advice (e.g., being aware of facilities available and finding physical activity partner). | The control group did not receive any intervention. |
| Roberts et al [40], 2019  [Wearable activity tracker] | All participants complete the initial eight-week exercise intervention. At the end of the eight-week intervention participants were given the goal to achieve 150-minutes of moderate-to-vigorous physical activity per week for the remaining 12 weeks of the study.  *Structured exercise program (8-weeks):* consists of a brief warm up, 30 minutes of moderate-intensity walking, 30 minutes of light lower- and upper-body resistance training, and balance and stretching exercises. Intensity was determined by the Borg category ratio 10 scale and heart rate monitor (Polar Ft2, Lake success, NY). Participants were instructed to walk a moderate intensity (5-6) with periods of vigorous walking (7-8).  *Cognitive behavioural training (8-weeks):* focused on reducing sedentary behaviour and increasing non-exercise physical activity in their daily life. Session occurred at the beginning of the intervention and before each of the exercise program sessions. Counselling was individualized to promote strategies participants could implement.  *Wearable activity tracker:* participants in the intervention group were provided a Fitbit Zip (San Francisco, CA, USA) and instructed to wear it during all waking hours except when at an exercise program session. The team tracked non-exercise physical activity provided additional motivation and individual based goals weekly at exercise program session and then over the phone for the remaining 12 weeks. Participants were instructed to record any time without wearing the tracker in a log given to them. | All participants complete the initial eight-week exercise intervention. At the end of the eight-week intervention participants were given the goal to achieve 150-minutes of moderate-to-vigorous physical activity per week for the remaining 12 weeks of the study.  *Structured exercise program (8-weeks):* consists of a brief warm up, 30 minutes of moderate-intensity walking, 30 minutes of light lower- and upper-body resistance training, and balance and stretching exercises. Intensity was determined by the Borg category ratio 10 scale and heart rate monitor (Polar Ft2, Lake success, NY). Participants were instructed to walk a moderate intensity (5-6) with periods of vigorous walking (7-8).  *Cognitive behavioural training (8-weeks):* focused on reducing sedentary behaviour and increasing non-exercise physical activity in their daily life. Session occurred at the beginning of the intervention and before each of the exercise program sessions. Counselling was individualized to promote strategies participants could implement. |
| Tabak et al [36], 2014  [Multi-component] | All participants received usual care during the four-week intervention period; this typically consisted of medication and physiotherapy (e.g., group training sessions). The intervention group participated in a multi-component intervention, made up of two modules. This group also received two 90-minute self-management sessions given by a nurse practitioner on how to use the website and identify symptoms that indicate the presence of a coming exacerbation.  *Activity coach:* collected information and provided feedback using an accelerometer (MTx-W sensor, Xsens Technologies, Enschede, The Netherlands) and a smartphone (HTC P3600/3700) both worn on the participant’s belt (connected to each other via Bluetooth). After the first week (used as baseline) participants began receiving feedback on changing their behaviour.  *Visual feedback:* the smartphone displayed a graph of the participants cumulative activity compared to a reference line (from 56 healthy controls). Participants were instructed to try and change the physical activity levels to match their line to that of the reference.  *Text message feedback*: based on the difference between lines participants were automatically sent a text message. These contained a summary of their activity behaviour and advice on how to improve or maintain their activity behaviour.  *Website:* participants were asked to complete an electronic diary every day. Which the website would then use to propagate advice to whether they should start medication in the case of an exacerbation | All participants received usual care during the four week intervention period; this typically consisted of medication and physiotherapy (e.g., group training sessions). |
| Talbot et al [44], 2003  [Pedometer] | All participants attended an arthritis self-management education. The education session was 12 hours and run by one of two registered nurses who attended a 16-hour arthritis foundation’s training course. Participants were taught techniques for coping with pain, in particular 1-hour was spent of exercise and arthritis self-management. Participants in the intervention group also received the Walk+ program.  *Pedometer:* a registered nurse instructed participants to wear the pedometer (New Lifestyles Digi-walker SW-200, Yamax, Tokyo, Japan) fastened to the side of the belt for daily step count monitoring.  *Activity logs:* participants were instructed to record their daily step counts and weekly averaged to determine if participants met goals. They were instructed to bring their logs and bring to their counselling sessions.  *Counselling:* A brief in person counselling session (< 5-minutes) was used to review activity logs and provide feedback. Every 4 weeks step count goals were increased by 10%, creating a 30% increase from baseline at the end of the 24-week intervention. | All participants attended an arthritis self-management education. The education session was 12 hours and run by one of two registered nurses who attended a 16-hour arthritis foundation’s training course. Participants were taught techniques for coping with pain, in particular 1-hour was spent of exercise and arthritis self-management. |
| Weinstock et al [42], 2011  **Smart-technology alone*  [Multi-component] | Participants were enrolled in the IDEATel study for five years (time periods ranged from 2000-2007 as participant were recruited for the first two years) of the. The intervention group received augmented care through a telemedicine intervention.  *Home telemedicine unit* (HTU): The HTU (American  Telecare Inc., Eden Prairie, Minnesota) is a web enabled computer that plugs into an existing phone line and provides four different services to the participants: 1) videoconferencing, 2) self-monitoring of fingerstick glucose and blood pressure, 3) messaging, and 4) web access.  *Video conferencing:* participants completed a videoconference with a diabetes educator every four to six weeks. They reviewed self-management education, home blood glucose and blood pressure measurement, and individualized goal setting.  *Pedometer*(added in 2004): all participants received a pedometer with written instructions. Diabetes educators instructed participants on proper use of the pedometer. If appropriate they else assisted in goal setting; initial goals were to wear the pedometers, followed by ten percent increases in steps a day or maintaining. Participants were instructed to write down daily step counts and share during video visits. | Participants in the control group received usual care from primary care provider.  *Pedometer* (add in 2004): all participants received a pedometer with written instructions. The control group was instructed to ask their primary care provider to advise them on goal setting and safe use of pedometers. The primary care providers set goals with their patients as they deemed appropriate. |
| Yates et al [45], 2009  [Pedometer] | All participants in both intervention groups received the  Prediabetes Risk Education and Physical Activity Recommendation and Encouragement (PREPARE) program, with two different methods of physical activity monitoring.   - PREPARE program with pedometer - PREPARE program without pedometer (time-based)   *PREPARE program:* the program was delivered by two educators trained through the Diabetes Education and Self- Management for Ongoing and Newly Diagnosed program. There was a single-session of group education; 105minutes covering causes, complications, timeline and identity of impaired glucose intolerance and 75-minutes effectiveness of exercise as treatment, walking self-efficacy beliefs, barriers to walking, and self-regulatory strategies.  *Progress review:* during their three- and six-month clinical measurement sessions all participants also received a ten-minute review of progress by the same educators.  *Pedometer:* participants in one group were provided with a pedometer and encouraged to set personalized steps per day goals based on their baseline activity levels.   - Sedentary participants were encouraged to increase their activity levels by at least 3000 steps per day (equivalent to ~30 min of walking). - >6000 steps per day were instructed to try and reach at least 9000. - Participants reaching >9000 steps per day were encouraged to maintain activity levels   Goal achievement was facilitated using proximal objectives dictating when, were and how; for example, achieving a 500 step a day increase every two weeks. Participants were instructed to wear pedometer daily and use a steps per day log to monitor their progress.  *Time based goals:* The second group did not receive a pedometer instead participants were instructed to set time-based goals, designed to match the pedometer group.   - Sedentary participants were encouraged achieve at least 30 min of moderate intensity walking per day. - Participants already walking 30 minutes a day were instructed to maintain this level and informed of the benefits of increasing activity levels further.   Participants set proximal goals; for example, increasing daily moderate intensity activity by five minutes every two weeks. Recommendations were also made to form action plans and record daily activity. (participants wanting to set goals for vigorous intensity activity were instructed to consult their general practitioner before beginning the program). | Participants in the usual care group received an information sheet in the mail. Information included likely causes, consequences, symptoms, and timeline associated with impaired glucose intolerance. There was also information on physical activity and its role in treating and controlling impaired glucose intolerance. |

# 4. RISK OF BIAS SUMMARY

## Figure 1. Risk of bias of all included studies


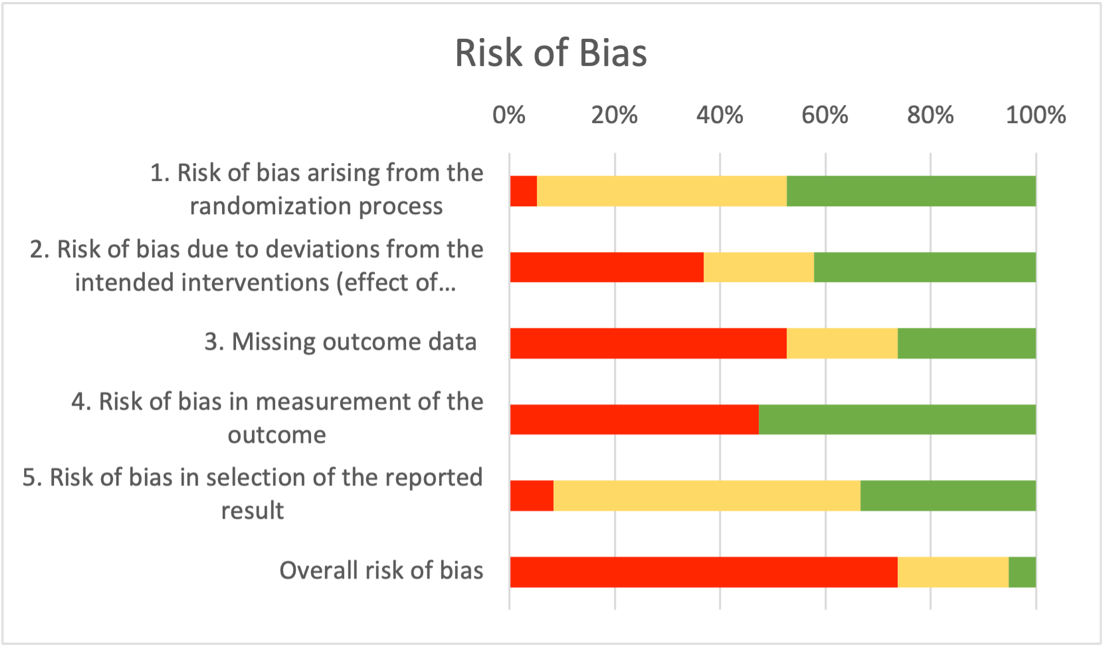


# 5. PHYSICAL ACTIVITY META-ANALYSES

## Figure 2. Steps per day subgroup analyses

## Figure 3. Total PA subgroup analyses

## Figure 4. Sensitivity Analyses

# 6. PHYSICAL FUNCTION META-ANALYSES

## Figure 5. The 6MWT subgroup analyses

## Figure 6. The 30-second sit-to-stand subgroup analyses

## Figure 7. Sensitivity analyses

# 7. SECONDARY OUTCOME META-ANALYSIS

## Figure 8. Depression meta-analysis

## Figure 9. HRQoL meta-analyses
